# The biogeochemical variability of Arctic thermokarst ponds is reflected by stochastic and niche‐driven microbial community assembly processes

**DOI:** 10.1111/1462-2920.15260

**Published:** 2020-10-13

**Authors:** Alizée Le Moigne, Maciej Bartosiewicz, Gabriela Schaepman‐Strub, Samuel Abiven, Jakob Pernthaler

**Affiliations:** ^1^ Limnological Station, Department of Plant and Microbial Biology University of Zurich Zürich Switzerland; ^2^ URPP Global Change and Biodiversity University of Zürich Zürich Switzerland; ^3^ Department of Environmental Sciences University of Basel Basel Switzerland; ^4^ Department of Evolutionary Biology and Environmental Studies University of Zurich Zürich Switzerland; ^5^ Department of Geography University of Zurich Zürich Switzerland; ^6^ Laboratoire de Géologie, UMR 8538 Ecole Normale Supérieure, CNRS PSL Research University Paris France; ^7^ Centre de Recherche en Ecologie Expérimentale et Prédictive (CEREEP‐Ecotron IledeFrance), Département de Biologie, Ecole Normale Supérieure, CNRS PSL Research University Paris France

## Abstract

Shallow thermokarst ponds are a conspicuous landscape element of the Arctic Siberian tundra with high biogeochemical variability. Little is known about how microbes from the regional species pool assemble into local pond communities and how the resulting patterns affect functional properties such as dissolved organic carbon (DOC) remineralization and greenhouse gas (GHG) turnover. We analysed the pelagic microbiomes of 20 ponds in north‐eastern Siberia in the context of their physico‐chemical properties. Ponds were categorized as polygonal or trough according to their geomorphological origin. The diversity of bacteria and eukaryotic microbes was assessed by ribosomal gene tag sequencing. Null model analysis revealed an important role of stochastic assembly processes within ponds of identical origin, in particular for genotypes only occurring in few systems. Nevertheless, the two pond types clearly represented distinct niches for both the bacterial and eukaryotic microbial communities. Carbon dioxide concentration, indicative of heterotrophic microbial processes, varied greatly, especially in the trough ponds. Methane concentrations were lower in polygonal ponds and were correlated with the estimated abundance of methanotrophs. Thus, the overall functional variability of Arctic ponds reflects the stochastic assembly of their microbial communities. Distinct functional subcommunities can, nevertheless, be related to GHG concentrations.

## Introduction

Lakes and ponds are common landscape elements of the arctic permafrost lowlands. The most prevalent water bodies of the northern tundra are small shallow ponds with a surface area under 1 km^2^ and a depth lower than 2 m (Verpoorter *et al*., 2014; Muster *et al*., 2017). These small ponds are disappearing in some regions like the Alaskan North slope (Riordan *et al*., 2006; Andresen and Lougheed, 2015), while expanding in size and numbers in others, such as subarctic Canada (Payette, 2004) and north‐eastern Siberia (Walter *et al*., 2006). Although the hydrology of these ponds seems to follow natural cycles of formation, expansion and drainage (van Huissteden *et al*., 2011), climate warming is likely to trigger an acceleration of these cycles in the Arctic regions (Vincent *et al*., 2013).

Given their shallowness and small size, arctic ponds are characterized by a large interface between the water and the sediments, the surrounding terrestrial ecosystems and the atmosphere. Although ice‐covered or frozen solid during most of the year, they are often nutrient‐rich and support high primary productivity (Przytulska *et al*., 2016) as well as active bacterial communities (Breton *et al*., 2009), highlighting their role as biogeochemical hotspots (Laurion *et al*., 2010; Deshpande *et al*., 2016). Arctic terrestrial ecosystems are particularly sensitive to climate change, resulting in the rapid thawing of the permafrost, thereby mobilizing the important stock (Tarnocai *et al*., 2009) of ancient and labile contemporary organic carbon (OC) into the shallow thermokarst ponds (Schuur *et al*., 2015). Although periodically acting as CO_2_ sinks (Laurion *et al*., 2010), thermokarst ponds are often CO_2_ and CH_4_ oversaturated and thus generally recognized as a significant source of greenhouse gases (GHGs) (Sobek *et al*., 2005; Breton *et al*., 2009). CH_4_ emissions from such ponds account for two‐third of the total emissions in regions above the latitude 50°N (Wik *et al*., 2016) and are strongly stimulated by atmospheric warming (Negandhi *et al*., 2014; Negandhi *et al*., 2016; Bartosiewicz *et al*., 2019). However, shallow ponds are still mostly neglected in estimates of regional and global C budgets (Muster *et al*., 2012).

Ponds in the Arctic originate from various geomorphological processes (Pienitz *et al*., 2008) that can lead to diverse biogeochemical properties (Breton *et al*., 2009). For instance, polygonal ponds form during the freeze–thaw cycles of ice wedges (Lachenbruch, 1962) and display transparent waters with benthic microbial mats (Bonilla *et al*., 2005). Other ponds form in depressions created by the underlying thawing permafrost and display turbid waters containing high amounts of dissolved OC (DOC) (Rautio *et al*., 2011). Shallow ponds also show remarkable spatio‐temporal variability in their GHG emissions (i.e. CH_4_ emissions ranging from 0.03 to 5.62 mmol m^−2^ day^−1^; Laurion *et al*., 2010). Although DOC concentration is an important factor controlling GHG emissions (Breton *et al*., 2009), the reasons for such disparities remain poorly understood.

The biogeochemical cycle of C in aquatic systems is mainly driven by microbial activity (Del Giorgio and Cole, 1998). The rates of C remineralization and storage depend on the interplay of abiotic conditions, such as pH and temperature (Muscarella *et al*., 2019). At the same time, it is unclear to which extent the microbial community composition and process rates actually reflect the local environmental conditions (Langenheder *et al*., 2006; Graham *et al*., 2016). In view of stochastic community assembly processes such as dispersal and ecological drift, the specific traits of individual microbial taxa may not be decisive for their respective occurrence patterns (Stegen *et al*., 2013; Langenheder *et al*., 2017). The study of β‐diversity allows to assess the link between the environment, biodiversity and ecosystem functioning. In this context, null model approaches are powerful tools to assess the importance of various community assembly processes. The Raup‐Crick (RC) metric represents a significance test that allows to disentangle changes in β‐diversity from variation in α‐diversity (Chase *et al*., 2011) while the normalized stochasticity ratio (NST) is an index that has been developed to quantify the relative importance of deterministic and stochastic community assembly processes (Ning *et al*., 2019).

While microbial community composition across permafrost thawing gradients in the Canadian Arctic appears to be mainly shaped by environmental filtering (Crevecoeur *et al*., 2015), stochastic community assembly processes can be dominant in ponds formed on organic permafrost (Comte *et al*., 2016). So far, only few studies have sought to integrate the variability of microbial community structure with that of physico‐chemical properties to assess the role of small thermokarst ponds in the C cycle (Vonk *et al*., 2015).

We investigated the bacterial and eukaryotic pelagic microbial communities in the context of physico‐chemical variability in two distinct types of shallow Arctic ponds with the following objectives: assess (i) if the microbial community structure in these ponds was primarily shaped by neutral assembly processes or determined by differences in habitat structure and/or pond origin and (ii) if the microbial assembly processes and resulting structure were reflected in the biogeochemistry of these shallow ponds in the context of GHG saturation and potential emissions.

## Results

### Morphometric properties and surrounding vegetation

**Fig. 1:**
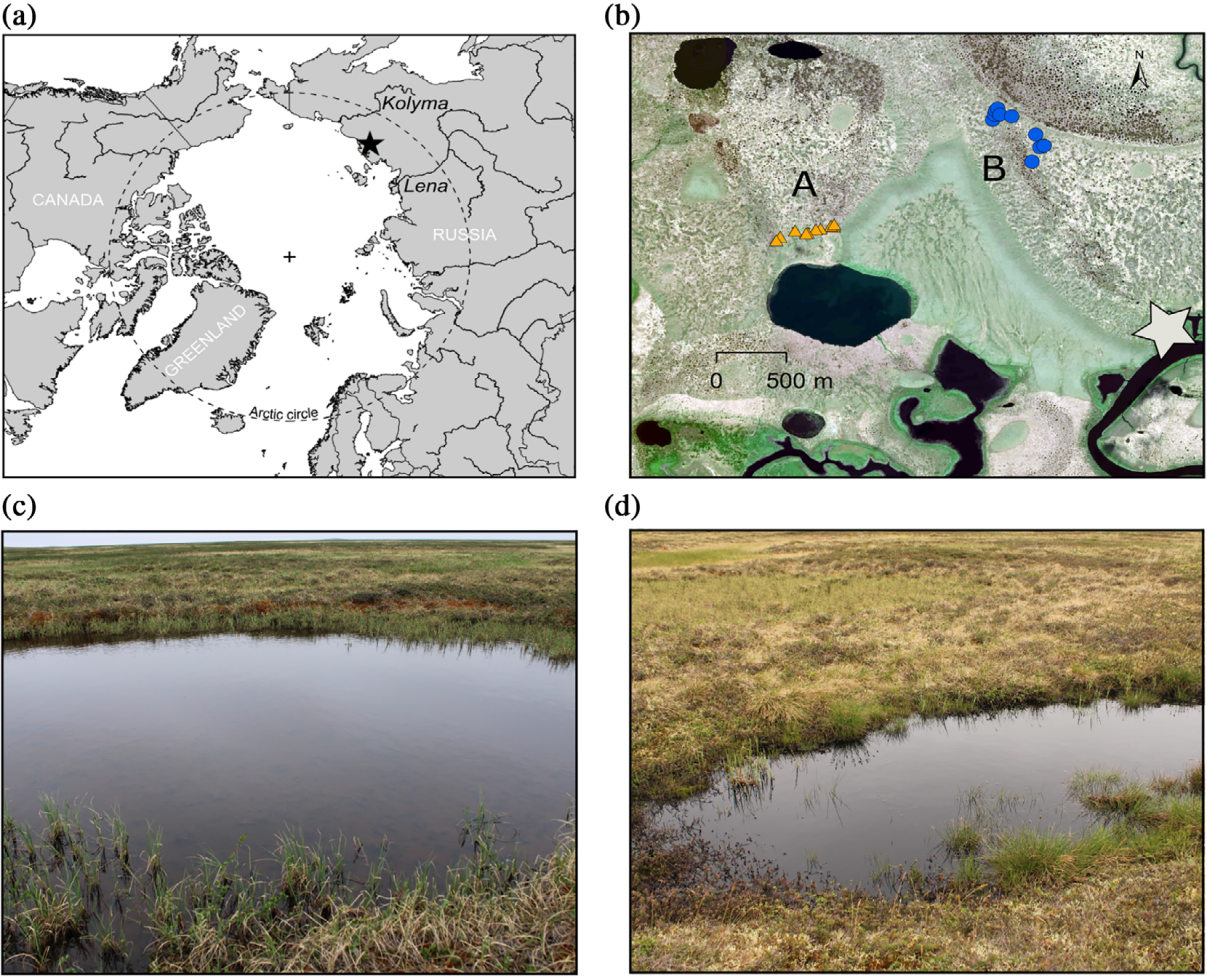
a. The Kytalyk region in North‐Eastern Siberia, Russia. b. Location of the two sampling sites, situated 1.5 km apart. The star represents the camp. c. Example of a polygonal pond at site A. d. Example of a trough pond with flooded vegetation at site B.

All ponds were small with a surface area not exceeding 150 m^2^ (Fig. 1, Fig. S1). The polygonal ponds (site A) were on average larger than the trough ponds (site B) (Table 1) with large variability between the ponds. All ponds were very shallow, their depth ranging between 20 and 30 cm (Table 1). The active layer at the bottom of the ponds ranged from 24 to 40 cm, while the active layer in the neighbouring soil was between 17 and 30 cm. The two types of ponds were surrounded by different plant species (Fig. S2). The dominant vegetation around the polygonal ponds was *Sphagnum fuscum* and *Carex aquatilis*, while the trough ponds were surrounded by *Ledum palustre*, *Eriophorum vaginatum*, *Betula nana* and the moss *Dicranum*. The trough ponds were characterized by the presence of submerged *Betula nana* (Fig. 1D).

**Table 1:** Comparison of morphometric characteristics and physico‐chemical properties of the 10 polygonal (site A) and 10 trough (site B) ponds (means ± standard deviation).

Property	Polygonal (A)	Trough (B)	*p*‐value
Elevation (m)	17.7 ± 0.92	13.5 ± 0.69	**0.0007**
Surface area (m^2^)	144.3 ± 100.4	57.9 ± 55.7	**0.03**
Depth (cm)	22 ± 2.5	23 ± 4.1	n.s.
Active layer depth (cm)	34.3 ± 1.9	33.8 ± 4.9	n.s
pH	5 ± 0.1	5 ± 0	n.s
Temperature (°C)^a.^	15.0 ± 1.1	15.9 ± 1.2	n.s
DOC (mg l^−1^)	21.54 ± 5.13	62.02 ± 18.44	**0.0002**
DN (mg l^−1^)	0.01 ± 0.02	0.95 ± 0.41	**0.0007**
DIC (mg l^−1^)^a.^	0.62 ± 0.39	0.23 ± 0.20	n.s
cDOM a_320_ (m^−1^)	41.06 ± 16.6	170.97 ± 58.5	**0.0003**
SUVA_254_ (l mg^−1^ m^−1^)^a.^	1.98 ± 0.40	2.81 ± 0.38	**0.0009**
Slope ratio	3.96 ± 0.15	3.68 ± 0.07	**0.0006**
POC (mg l^−1^)	2.45 ± 1.29	5.82 ± 4.16	**0.02**
δ ^13^C POC (‰)	−32.3 ± 2.05	−32.6 ± 1.95	n.s
dissolved CO_2_ (μM)	11.61 ± 10.24	26.62 ± 25.10	n.s
CO_2_ saturation (%)	65.7 ± 58	150.6 ± 142	n.s.
dissolved CH_4_ (μM)	0.76 ± 0.61	5.99 ± 7.78	**0.006**

a. Comparisons of these mean values were performed with 2‐sided*t*‐tests. The other parameters were compared by nonparametric Mann–Whitney U‐tests.

Significant differences are highlighted in bold. DOC: dissolved organic carbon, DN: dissolved nitrogen, DIC: dissolved inorganic carbon, cDOM a_320_: absorption coefficient by chromophoric dissolved organic matter at 320 nm, SUVA254: specific UV absorbance at 254 nm, POC: particulate organic carbon.

### Physico‐chemical characteristics

Chlorophyll‐a (Chl‐a) concentrations were on average 1.1 μg l^−1^ (range 0.2 to 5.2 μg l^−1^) across all ponds (excluding pond B05 that had an exceptionally high Chl‐a concentration of 38.1 μg l^−1^), without differences between the two types (Mann–Whitney, *W* = 54, *p* = 0.5). DOC and cDOM were three and four times higher, respectively, in the trough ponds than in the polygonal ponds (Table 1). The trough ponds were also enriched in POC and DN compared to the polygonal ones, while the latter contained slightly more DIC. The DOC of the trough ponds consisted of more aromatic (higher SUVA_254_) and higher molecular weight compounds (lower slope ratio) than that of the polygonal ponds. The δ^13^C isotopic signal of POC averaged −32‰ in both pond types. Differences in carbon content were also visible by colour: the trough ponds were dark brown, while the polygonal ponds were transparent. Ponds from both sites displayed pronounced variability in their water isotopic signatures (Fig. S3), and their local evaporation line deviated from the global meteoric water line. The water of all ponds was enriched in heavy water isotopes, with polygonal ponds being significantly more enriched in deuterium (*t*‐test, *t* = 3.8, *df* = 18, *p* = 0.004) but not in ^18^O (*t*‐test, *t* = 1.6, *df* = 18, *p* = 0.15). The ponds were acidic with a pH of five and the water temperature was uniform over the sampling period. The amount of dissolved CO_2_ in the ponds varied greatly and oversaturation (CO_2_ equilibrium at 15.5°C: 17.7 μM) was found in five trough ponds and one polygonal pond, while the others were CO_2_ depleted (Table 1). All ponds were oversaturated in dissolved CH_4_ compared to atmospheric concentrations (CH_4_ equilibrium at 15.5°C: 3.1 × 10^−3^ μM). Dissolved methane concentrations were highly variable, ranging from 0.18 μM to 27.5 μM, and they were significantly higher in the trough ponds (Table 1). The δ^13^C‐CH_4_ signatures varied among the polygonal (mean −48.9‰, range −58.4‰ to −30.3‰) and the trough ponds (mean −49.0‰, range −69.8‰ to −16.5‰). The range of variability was somewhat larger in the trough ponds but this difference was not statistically significant (*F* test, *F* = 0.28, *p* = 0.11). The water isotopic signal (δ^2^H) was correlated with the CH_4_ concentration (Spearman's *ρ* = −0.51, *p* = 0.04) as well as with the δ^13^C‐CH_4_ signatures (Pearson's *r* = 0.48, *p* = 0.04).

### Microbial community composition

**Fig. 2:**
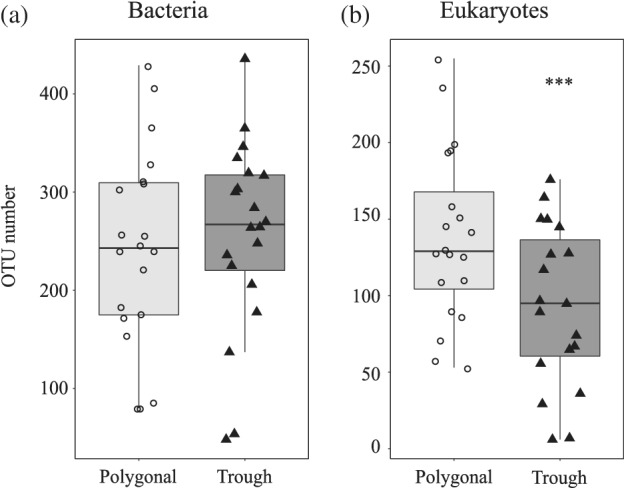
Number of OTUs in ponds from the two sites: (a) bacterial communities, (b) microbial eukaryotic communities. Asterisks indicate significant differences at *p* < 0.001.

Bacterial abundances ranged from 3.9 to 10.3 × 10^6^ cells ml^−1^ in the polygonal ponds and from 4.8 to 29.1 × 10^6^ cells ml^−1^ in the trough ponds, with no significant difference between pond types (median values for polygonal ponds: 6.1 × 10^6^ cells ml^−1^, trough ponds: 7.4 × 10^6^ cells ml^−1^, Mann–Whitney, *W* = 34, *p* = 0.34). Bacterial OTU numbers in the normalized data set varied from 60 to 420 OTUs (Fig. 2A), without difference between pond types (*t*‐test, *t* = −0.46, *df* = 38, *p* = 0.64). Bacterial abundances and OTU numbers did not correlate with DOC (respectively: Spearman's *ρ* = 0.23, *p* = 0.3; Spearman's *ρ* = 0.13, *p* = 0.5). OTU numbers of eukaryotic microbes ranged from 6 to 255 OTUs (Fig. 2B), with polygonal ponds being more diverse than the trough ponds (*t*‐test, *t* = 2.57, *df* = 38, *p* = 0.01). Pond B05 displayed the lowest diversity of both, bacteria and microbial eukaryotes.

**Fig. 3:**
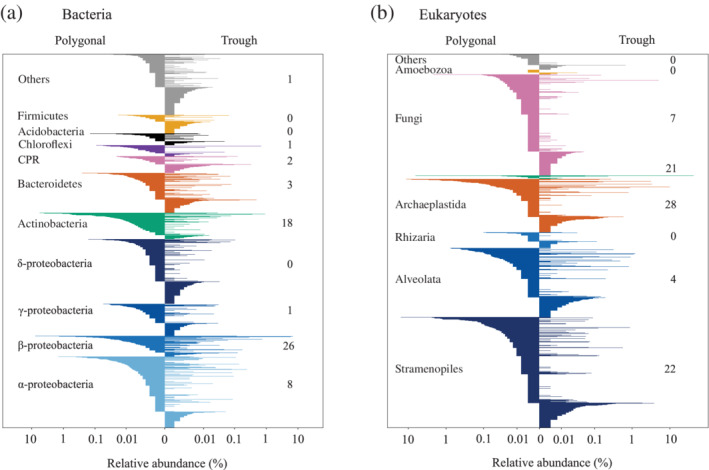
OTU composition within different phylogenetic lineages of the (a) bacterial and (b) microbial eukaryotic communities of polygonal ponds and trough ponds. OTUs in the polygonal ponds (left side of panels) were sorted according to their contribution to all OTUs in this pond type (*y*‐axis), and plotted against their corresponding contribution in the trough ponds (right side of panels). The numbers in the right half of the panels report the relative contribution of individual phylogenetic lineages to the average dissimilarity between polygonal and trough ponds communities (SIMPER analysis of Bray‐Curtis distances, significantly contributing OTUs only).

Bacterial communities in both pond types were dominated by *Proteobacteria*, *Actinobacteria* and *Bacteroidetes* (Fig. 3A). The most ubiquitous and numerous OTUs were a sequence affiliated to the genus *Polynucleobacter* and an OTU that matched 100% to database entries assigned to both, *Variovorax* (accession number: MT102304.1) and *Rhodoferax* (accession number: MH699237.1) in BLAST. We also noted the presence of OTUs affiliated to the Candidate Phyla Radiation. Similarity percentage analysis (SIMPER) indicated that OTUs affiliated with *Variovorax/Rhodoferax*, *candidatus Planktoluna*, *Limnohabitans* and *Rhodoblastus* preferably inhabited the trough ponds while an uncultured *Burkholderiaceae* (with a similar sequence to *Alcaligenes)*, *Rhodoluna*, *candidatus Planktophila* (acl‐A) and *Methylorosula* were more common in the polygonal ponds (Table S1).

**Fig. 4:**
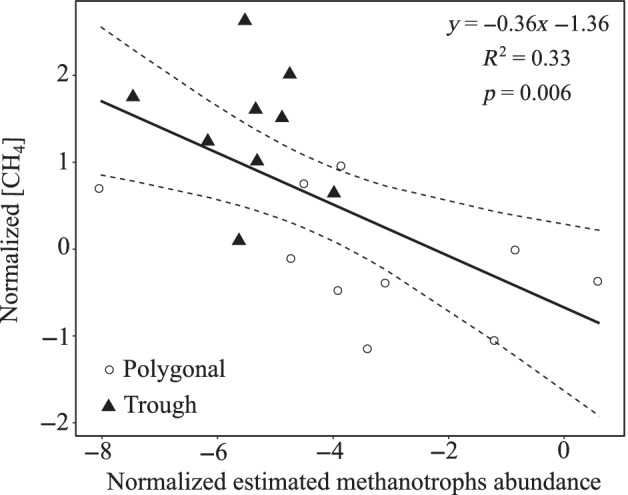
Relationship between the estimated abundance of methanotrophs and dissolved CH_4_ concentration. Data were normalized by a Box‐Cox transformation (*λ* = −0.14). Pond B05 was excluded from the analysis due to its lack of methanotroph reads (see text).

Methanotrophs belonged to the genera *Methylocapsa*, *Methylocystis*, *Methylosinus*, *Methylocella*, *Methylobacterium*, *Methylococcus*, *Crenothrix*, *Methylomonas*, *Methyloparacoccus*, *Methyloglobulus* and *candidatus Methylospira* (Fig. S4). On average, 1.2% (range 0.02–9.1%) of the bacterial reads in the polygonal ponds and 0.2% (range 0–0.9%) in the trough pond were affiliated with these taxa, with some indication of differences in their community composition between pond types (Fig. S4). More methanotrophs (estimated abundance) were present in polygonal ponds (Mann–Whitney, *W* = 334, *p* < 0.001). Their estimated abundance was moreover negatively correlated with dissolved methane concentrations (Spearman's *ρ* = −0.63, *p* = 0.01) (Fig. 4) and positively correlated with the δ^13^C‐CH_4_ signatures (Spearman's *ρ* = 0.51, *p* = 0.04).

The eukaryotic microbial communities in both pond types mainly consisted of members of the SAR (*Stramenopiles*, *Alveolata*, *Rhizaria*) lineage, *Archaeplastida*, *Cryptophyceae* and fungi (Fig. 3B). The difference between the communities of the polygonal and trough ponds (SIMPER) was mainly due to members of *Archaeoplastida* (genera *Monomastix*, *Chaetosphaeridium*) and *Stramenopiles* (*Uroglena*) that were more abundant in the polygonal ponds (Table S2). The trough ponds had higher proportions of reads from a single OTU that was 100% identical to *Cryptomonas marssonii* and that accounted for 21% of the separation between pond types. Pond B05 (which also exhibited unusual high Chl‐a concentrations, see above) was dominated by a genotype with 98.4% sequence identity with a *Chlamydomonas proboscigera* (GU117581) isolated from Svalbard. Fungi were also an important component of the eukaryotic communities, mainly represented by the class *Chytridiomycetes*.

### β‐diversity of microbial communities across ponds

**Fig. 5:**
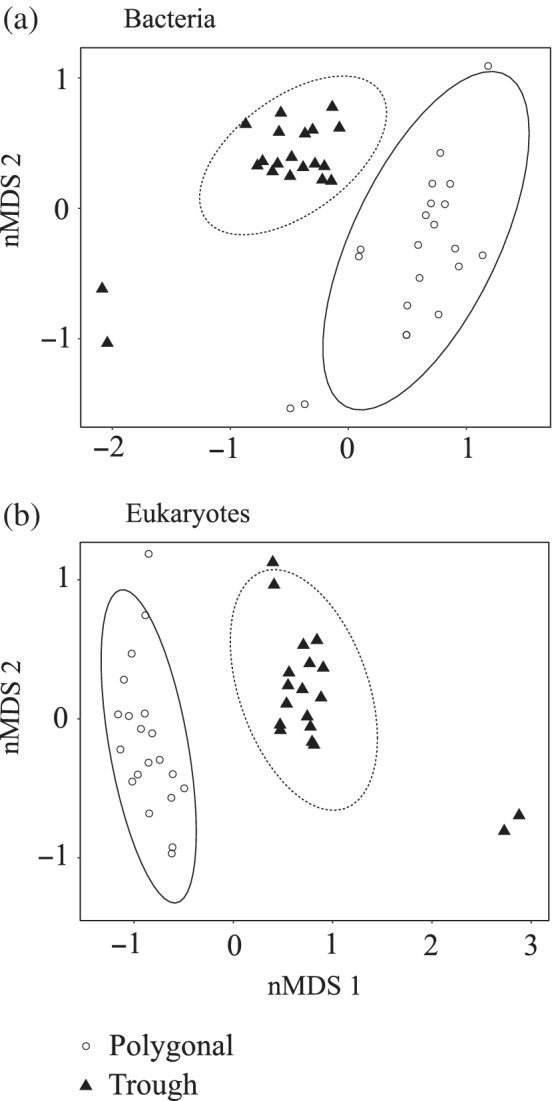
Non‐metric Multidimensional Scaling (nMDS) of (A) the bacterial communities (stress = 0.15) and (B) the eukaryotic microbial communities (stress = 0.14) based on Bray‐Curtis β‐diversity. The ellipses represent the 95% confidence interval of the position of each group.

Within each pond type, the average pairwise Bray‐Curtis (BC) similarity of bacterial communities was lower than 50% (polygonal ponds: mean 33%, range 5–78%; trough ponds: mean 34%, range 3–80%). Both types of ponds showed similar variability in bacterial community structure (homogeneity of multivariate dispersions, N.perm = 9999, *F* = 0.007, *p* = 0.92). However, polygonal and trough ponds were composed of distinct communities (PERMANOVA, N.perm = 9999, *F* = 6.9, *R* ^2^ = 0.16, *p* < 0.001) (Fig. 5A), with low pairwise similarity between ponds of different types (mean 25%, range 3–77%).

The BC similarity of the eukaryotic microbial communities was low within polygonal ponds (mean 19%, range 2–51%), whereas trough ponds were on average more similar to each other (mean 71%, range 0–90%). The variability of eukaryotic community composition was similar for both pond types (homogeneity of multivariate dispersions, N.perm = 9999, *F* = 0.88, *p* = 0.36). Nevertheless, the microbial eukaryotic communities significantly differed between pond types (PERMANOVA, N.perm = 9999, *F* = 9.6, *R* ^2^ = 0.20, *p* < 0.001) (Fig. 5B), with very low similarity between pond pairs from different types (mean 0.07%, range 0–37%).

### Microbial community assembly processes

Stochastic processes dominated the assembly of bacterial communities within both pond types, as indicated by NSTs > 50% and more than 80% of RC indices ranging between −0.95 and 0.95 (Table 2). The contribution of stochastic processes was similar in both pond types (bootstrap test on NST, *p* = 0.39). However, two lines of evidence suggested that the two sets of ponds represented distinct niches: their between‐type NST was < 50%, and approximately 30% of the between‐type RC comparisons were less similar than expected by chance. There was no clustering nor over‐dispersion of phylogenetic diversity either within or between the two sites [−2 < Beta Nearest Taxon Index (βNTI) < 2].

**Table 2:** Community assembly processes of polygonal ponds (site A) and trough ponds (site B) as assessed by null model based indices.

Bacteria	Polygonal (A)	Trough (B)	A versus B
NST	63.4	65.4	43.9
RC < −0.95	2.3	2.3	2
−0.95 < RC < 0.95	84.4	97.7	63
RC > 0.95	13.3	0	35
βNTI	−1.44	−0.82	−1.10

Eukaryotes	Polygonal (A)	Trough (B)	A vs. B
NST	50.6	37.5	30.2
RC < −0.95	0	13.3	0
−0.95 < RC < 0.95	64.4	84.4	38
RC > 0.95	35.5	2.2	62
βNTI	−0.22	−0.38	−0.54

βNTI: Beta Nearest Taxon Index, NST: normalized stochasticity ratio (%), RC: modified Raup‐Crick (% of pairwise comparisons within specified range). Polygonal, trough: within sites; A versus B: between sites.

**Fig. 6:**
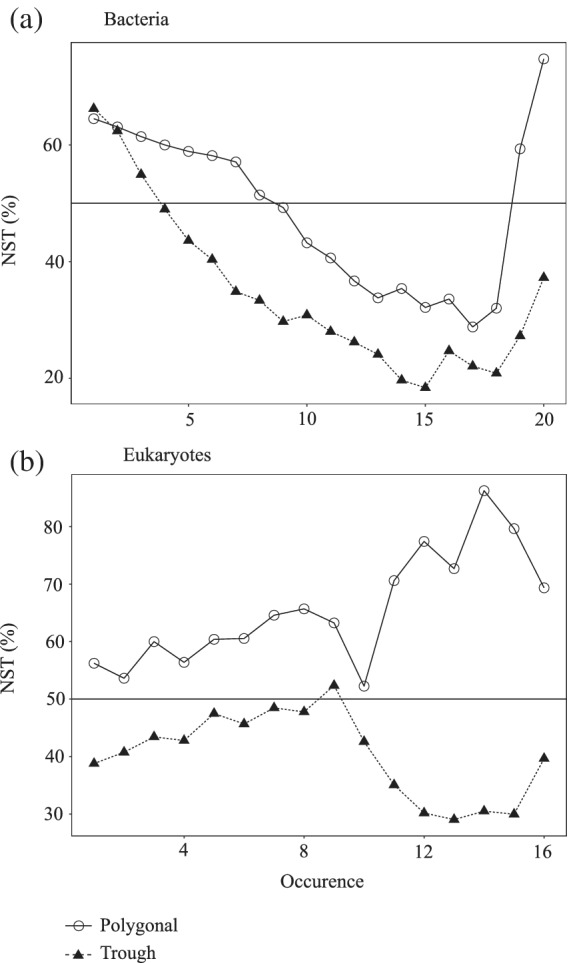
Normalized stochasticity ratio (NST) of subsets of OTUs occurring in increasing numbers of ponds (*n*_max_ = 20 and 16 ponds for bacteria and eukaryotes respectively).

The community assembly processes of the eukaryotic microbial community appeared to be more related to the pond type. Specifically, stochastic processes dominated in the polygonal ponds and deterministic processes in the trough ponds (Table 2). However, this difference was not statistically significant (bootstrap on NST, *p* = 0.07). Interestingly, 35% of polygonal pond pairs were less similar than expected by chance (RC > 0.95), whereas the opposite appeared in the trough ponds, with 13% of the pairs being more similar than expected by chance (RC < −0.95). Pond type determined the microbial eukaryotic communities even stronger than the bacterial communities (NST_A vs. B_ = 30%), and communities originating from different pond types were less similar than expected by chance in 62% of the RC comparisons. The phylogenetic composition of the eukaryotic microbial community was neither clustered nor over‐dispersed within and between the two types of ponds (−2 < βNTI < 2). The NST indices of bacterial communities from both pond types steeply decreased if only the more widely distributed OTUs were considered (Fig. 6), and it rose again for the small subset of the most ubiquitous OTUs that were present in 18 ponds or more. No such trend was observed for eukaryotic microbes, and no eukaryotic OTU occurred in more than 16 of the ponds.

The influence of deterministic processes on bacterial community composition was also indicated by a weak but significant correlation with the measured environmental variables (Mantel test, 9999 permutations, Spearman's *ρ* = 0.20, *p* = 0.04). The distance‐based redundancy analysis (db‐RDA) (Fig. S5A) was significant (*df* = 3, *R* ^2^ = 0.35, *p* = 0.001) with DOC, DIC and δ^13^C POC as the main explanatory variables and explained nearly 50% of the total inertia (CAP 1: 36%, CAP 2: 12%). The microbial eukaryotic communities were also significantly correlated with the environmental variables (Mantel test, 9999 permutations, Spearman's *ρ* = 0.51, *p* < 0.001). The db‐RDA (Fig. S5B) was also significant (*df* = 7, *R* ^2^ = 0.28, *p* = 0.001) with DOC, DIC and POC as the main explanatory variables. Only the CAP 1 explained inertia (21%).

## Discussion

### Pond types differ in habitat properties

The shallow ponds of the Kytalyk region, although closely located to each other, were remarkably variable in their biogeochemical properties (Table 1), as has also been observed in thermokarst ponds in other circumpolar regions (Breton *et al*., 2009; Shirokova *et al*., 2009; Rautio *et al*., 2011). The two pond types were set apart particularly by the quantity and optical properties of OC (DOC and POC). The OC in thermokarst ponds mainly originates from terrestrial input (Crump *et al*., 2003; Wauthy *et al*., 2018). It has a great influence on pond colour and turbidity, which in turn affects primary production and subsequent trophic processes (Fasching *et al*., 2014). The observed difference in DOC properties between pond types could partly be due to features of the catchment, such as the type of the adjacent vegetation or of soil, both known to influence the quantity and biodegradability of allochthonous DOC (Blom *et al*., 2000; Larouche *et al*., 2015). Indeed, the surrounding vegetation clearly differed between the sites (Fig. S2). Since the ponds were situated within the beds of two ancient thaw lakes of different drainage age, it is conceivable that the composition of the active layer and permafrost in carbon and ice might differ as well. The geomorphological origin and maturation of thermokarst ponds also influence the amount of C discharged into ponds (Pokrovsky *et al*., 2011; Peura *et al*., 2020). The banks of trough ponds appeared to be less stable than of the polygonal ones, as indicated by the presence of flooded *Betula nana*.

Photolysis in Arctic aquatic systems can be an important process in modifying DOC optical properties and bioavailability (Vähätalo *et al*., 2003; Laurion and Mladenov, 2013), especially in summer where surface waters are exposed to a 24 h light regime. The low amount of cDOM in the polygonal ponds in combination with higher DIC concentrations suggests that DOC underwent more intense photochemical degradation in these systems than in the trough ponds. However, bacterial abundances and α‐diversity were similar in both types of ponds and did not correlate with DOC quantity and type. This stands in contrast to observations from subarctic ponds (Roiha *et al*., 2012) and boreal lakes (Logue *et al*., 2012) and suggests that bacterial abundance and α‐diversity were not primarily controlled by DOC availability.

### Microbial community assembly processes

The bacterial and eukaryotic microbial communities of polygonal and trough ponds clearly differed in composition (Fig. 5), and null model analyses suggested that these differences were predominantly driven by deterministic processes (Table 2). Since the pond types were distinguished by geographic location and habitat characteristics (Fig. 1, Table 1), two scenarios could explain this observation. For one, each type of ponds might have a different local species pool as a result of dispersal limitation, which may be relevant even at small spatial scales (< 1 km) (Martiny *et al*., 2011). On the other hand, some studies have argued that dispersal limitation is only relevant at larger spatial scales and in physically well‐separated landscapes (Green *et al*., 2004; Comte *et al*., 2016). Assuming a minor role of dispersal limitation, the observed occurrence pattern of taxa in different pond types would be best explained by niche‐driven processes. In the case of bacteria, this conclusion is also supported by db‐RDA analysis (Fig. S5), with DOC explaining an important part of the variance in community composition. However, an NST index close to 50% and the 63% of the RC indices consistent with the null model both indicate that stochastic processes still played an important role in shaping bacterial communities between pond types. Deterministic factors between pond types were more important in the micro‐eukaryotic assemblages (Table 2). However, the link with the measured environmental parameters was weaker (Fig. S5), indicating that the relevant niche parameters were not accounted for.

Stochastic processes dominated the assembly of bacterial communities within both pond types and of eukaryotic microbes in polygonal ponds. Priority effects (e.g. impact of colonization order) and ecological drift (e.g. random changes in relative abundances of species) are major stochastic processes (Zhou and Ning, 2017) that might play an important role in the community composition of such ponds. Ponds represent small habitat patches and are seasonally ephemeral (they freeze solid during winter). This speaks for frequent pond‐specific colonization events, favouring priority effects (Fukami, 2015).

In the trough ponds, the NST index of eukaryotic microbial communities pointed to a dominance of deterministic assembly processes, and 15% of the RC indices in these ponds were more similar than expected by chance (Table 2). Thus, eukaryotic microbes might have experienced species sorting by pond characteristics that were not explicitly determined in this study, such as limited light availability for phototrophs (Thrane *et al*., 2014) or a stronger top‐down‐control by daphnids (Zöllner *et al*., 2003; Sarnelle, 2005) that were only present in trough ponds (A.L.M., personal observation).

Community assembly processes may vary for rare and abundant taxa (Liu *et al*., 2015; Nino‐Garcia *et al*., 2016). We observed a clear decrease of the NST index in subcommunities of those bacterial OTUs that occurred in an increasing number of ponds (Fig. 6A). This suggests that the stochastic overall assembly pattern of the bacterial communities was primarily shaped by taxa that were only present in a single or a few systems. Bacteria are inoculated into the ponds mainly from the surrounding soil that acts as a reservoir for bacteria with the potential to thrive in freshwater environments (Crump *et al*., 2012). Assuming a low abundance of many of these taxa, both priority effects and ecological drift would arguably add to community stochasticity (Nemergut *et al*., 2013). By contrast, bacterial taxa occurring in 10–15 of the 20 ponds seemed to be assembled deterministically (Fig. 6A). Many dominant taxa of aquatic bacteria are relatively invariant at regional scales (Ostman *et al*., 2010). Dispersal by mass effect dynamics of abundant taxa (Urban, 2004) in combination with either an euryoecious or a stenoecious life style could thus lead to such deterministic patterns. The apparent return to stochasticity for OTUs occurring in almost all systems (Fig. 6A) may reflect a limitation of null model approaches if performed on a limited number of taxa (Chase *et al*., 2011). Only two bacterial OTUs were present in all ponds, one of them (*Polynucleobacter*, PnecC) being a well‐studied case of ecological diversification below the resolution of the rRNA gene (Hahn, 2003).

The changes in assembly patterns of the eukaryotic microbial communities at increasing frequency of occurrence did not show the same general trend as of bacteria (Fig. 6B), suggesting that fundamentally different mechanisms acted on rare and common bacteria and eukaryotic microbes. Eukaryotes in the surrounding soil might be less prone to colonize the ponds, as was reported for rivers and lakes (Crump *et al*., 2012). The most widely distributed OTU was affiliated with the cosmopolitan flagellate *Cryptomonas marssonnii* (Menezes and Novarino, 2003), more abundant in the turbid trough ponds (Mann–Whitney, *W* = 89, *p* = 0.005) and possibly favoured by its mixotrophic lifestyle (Saad *et al*., 2013).

### GHG concentrations

Unlike many arctic ponds and lakes (Sobek *et al*., 2005; Repo *et al*., 2007; Breton *et al*., 2009), the majority of the studied ponds, in particular the polygonal ones, were net autotrophic, i.e. their water column CO_2_ levels were below the equilibrium concentration. While the quantity of Chl‐a was similar between pond types, the water transparency of the polygonal ponds (Fig. 1) allowed for formation of epibenthic cyanobacterial mats (A.L.M., personal observation). Such mats have been reported to be responsible for the bulk of primary production in comparable systems (Bonilla *et al*., 2005) and might explain the observed CO_2_ depletion. On the other hand, differences in Chl‐a were insufficient to explain why half of the trough ponds were CO_2_ undersaturated, whereas the other half was oversaturated. All these ponds were highly turbid, likely preventing cyanobacterial mats to form. Alternatively, differences in CO_2_ saturation levels could be related to chemical weathering of carbonates (Pierre *et al*., 2019). In contrast to previous reports (Breton *et al*., 2009), dissolved CO_2_ concentrations could not be predicted from the amount of DOC, suggesting that net auto‐ or heterotrophy was controlled by carbonate weathering or biotic factors such as food‐web structure (Schindler *et al*., 1997). The wide range of CO_2_ saturation levels might also be the result of asynchronous temporal dynamics, i.e. alternating blooms of autotrophs and grazers might cause ponds to switch between periods of net autotrophy and heterotrophy (Laas *et al*., 2012). While a conspicuous bloom of *Chlamydomonas* in a single trough pond (B05) paralleled by pronounced CO_2_ depletion seems to support this notion, a time‐resolved monitoring of CO_2_ concentrations and food web structure in several ponds would be required to substantiate it.

All ponds were oversaturated with CH_4_, highlighting the importance of thermokarst ponds as GHG sources (Laurion *et al*., 2010; Negandhi *et al*., 2014). The positive relationship between the isotopic signature of methane and the estimated abundances of methanotrophs reflects the accumulation of methane with higher ^13^C content in ponds with more methanotrophs. Such an accumulation is indicative of methanotrophy, as methanotrophs preferentially incorporate light methane (Smith *et al*., 1991). Methanotrophy is also suggested by the decrease of CH_4_ concentrations with the estimated abundance of methanotrophs (Fig. 4). Our findings expand previous results where a similar relationship was described at the sediment – water column interface (Crevecoeur *et al*., 2017) by highlighting the potential role of pelagic methanotrophs in mitigating CH_4_ emissions. However, methanotrophs featured a sizable community only in polygonal ponds, with an exceptionally high contribution to total bacterial reads in one pond (A07, 9%).

In contrast to prior findings from thermokarst ponds (Crevecoeur *et al*., 2017), the dominant methanotrophs in the polygonal ponds were of Type II. Type I and type II methanotrophs are distinct in their methanotrophic metabolic pathway as well as in their phylogenetic affiliation (Lüke and Frenzel, 2011). Type II methanotrophs have been reported to be dominant in boreal wetlands (Dedysh, 2009) and in some western Siberian lakes (Osudar *et al*., 2016). Although type II methanotrophs tend to thrive at higher temperatures than type I (Graef *et al*., 2011; He *et al*., 2012), the factors controlling the dominance of one type over the other are not fully understood (Osudar *et al*., 2016).

The low contribution of methanotrophs to total bacterial read numbers in the trough ponds illustrates that CH_4_ concentration from particular pond type may be largely unaffected by microbial consumption processes in the water column. CH_4_ fluxes in freshwater systems are controlled by both, biological and physical processes, such as temperature and stratification (Yvon‐Durocher *et al*., 2014; Bartosiewicz *et al*., 2015; Bartosiewicz *et al*., 2016). The relationship between the CH_4_ concentrations and δ^2^H‐H_2_O suggests that physical processes, such as evaporation losses could affect the potential release of CH_4_ in our study systems.

## Conclusion

Shallow ponds across the Arctic Siberian tundra showed striking variability in their geochemical features and in their bacterial and eukaryotic microbial communities. While the geomorphological origin, habitat conditions and geographic location of these ponds clearly affected the occurrence of eukaryotic microbes as well as of the widely distributed bacterial taxa and methanotrophs, stochastic assembly processes shaped communities of ponds from the same type and geographic region. This stochasticity of assembly processes was reflected in the functional variability of the ponds, notably in their balance of net auto‐ versus heterotrophy and CH_4_ concentration. The overall relationship between planktonic methanotrophs and methane concentrations highlights the need for a better understanding of the habitat preference of methanotrophs to assess and predict carbon budgets in permafrost areas.

## Experimental procedures

### Study site

The field campaign took place from July 13 to 23 2018 at the Kytalyk Nature Reserve (70.82N, 147.47E), a low‐arctic tundra area in North‐East Siberia, Russian Federation. The research site within the reserve is located in the continuous permafrost region, in the Indigirka lowlands, 30 km north from Chokurdakh (Fig. 1). An extensive site description can be found in Juszak *et al*. 2016. The permafrost of the region is carbon‐ and ice‐rich, (Yedoma) (Grosse *et al*., 2013; Strauss *et al*., 2013). In total, 20 shallow ponds were sampled from two distinct geomorphological sites situated 1.5 km apart. At site A, we selected 10 ponds presumably formed by thermal ground contraction, creating low‐centred polygons that can be filled by precipitation (Lachenbruch, 1962) (subsequently referred to as polygonal ponds). At site B, we selected 10 ponds most likely formed by the thawing of the underlying permafrost ice, named trough ponds hereafter. Both ponds types are frequent in the region (Li *et al*., 2017). The ponds at each site, while located relatively close to each other, were selected according to their clear shore delimitation and their lack of direct hydrological connectivity. All ponds were sampled once over a period of 2 weeks. Pond B01 was sampled three times during the campaign to assess the variability of its physico‐chemical and biological properties (Fig. S6).

### Physico‐chemical characterization of the ponds

The elevation of each pond was recorded by a hand‐held GPS device. The surface area was digitized with the ArcGIS software from an orthomosaic of drone images with a resolution of 15 cm taken during the sampling period. Maximum depth and active layer thickness in and around each pond were determined with a graduated steel rod. pH was assessed with pH paper (Whatman, indicator paper 0–14) and temperature with an analogue thermometer. Duplicate surface water samples were collected from all ponds with a Van Dorn sampler and filtered through pre‐combusted (4 h at 550°C) glass fibres filters (Whatman GF/F, pore‐size: 0.7 μm). The filtrate was collected in pre‐combusted glass vials and used to measure DOC, dissolved inorganic carbon (DIC), dissolved nitrogen (DN), optical properties of the dissolved organic matter (DOM) and the stable isotopic composition of the water (δ^18^O and δ^2^H). The filters served to determine particulate organic carbon (POC) and its stable isotopic composition (δ^13^C). Filters and filtrates were stored frozen in the permafrost cellar (≤ −5°C) of the research station and remained frozen until analysis in the laboratory. DOC, DIC and DN were measured using a Formacs HT‐I TOC/TN analyser (Skalar) calibrated with potassium phthalate. Optical properties of DOM were determined by spectrophotometric scans from 200 to 800 nm at steps of 1 nm (UV 1601, Shimadzu). The chromophoric DOM fraction (cDOM) was quantified by measuring the absorption coefficient of the water at 320 nm (a_320_). The specific ultraviolet absorbance index (SUVA_254_) was used as a proxy for the proportion of aromatic compounds (Weishaar *et al*., 2003). The Slope Ratio is an indicator of the molecular weight of the cDOM and was calculated across the spectral bands 275–295 and 350–400 nm as described in (Helms *et al*., 2008). To determine the stable isotope composition of the water, prefiltered samples were filtered again through 0.1 um pore size PES filters (Yeti, Infochroma) and measured by Off‐Axis Integrated Cavity Output Spectroscopy (OA‐ICOS) using a Liquid Water Isotope Analyser (LWIA‐24‐EP, Los Gatos Research). To determine POC and δ^13^C‐POC, the filters were dried for 72 h at 65°C, weighted and cut in half, then combusted at 900°C and analysed by Combustion Module‐Cavity Ring Down Spectroscopy (Picarro Inc.). The POC concentration was weighted by the filtered volume, ranging between 100 and 250 ml. Dissolved CO_2_ and CH_4_ concentration were obtained with the headspace technique (Kling *et al*., 1992; Laurion *et al*., 2010; Dinsmore *et al*., 2013). Fourty millilitre of pond water was equilibrated with 20 ml of ambient air. Samples were shaken, equilibrated for 1 min and the headspace was sampled in an evacuated helium‐purged 20 ml glass vial. The headspace and ambient air were collected in pentaplicates for each pond. CO_2_ and CH_4_ concentrations were measured by a gas chromatograph equipped with an FID detector and a methanizer and recalculated to aqueous values according to Henry's law:

(1) $${\mathrm{Gas}}_{\left(\mathrm{aq}\right)}=K_{H}\times \text{pGas}$$

where K_H_ is the Henry's constant adjusted for the water temperature and pGas the partial pressure of the gas in the headspace. Because samples were equilibrated in air, δ^13^C‐CH_4_ signatures were corrected for minor atmospheric contribution and further analyses were carried out only for samples with sufficiently high concentrations (CH_4_ > 0.2 μM), which resulted in missing information for two polygonal ponds. This analysis was performed in a pre‐concentration system (PreCon2018, Thermoscientific) coupled to an isotope ratio mass spectrometer (Gasbench II – Delta V Advantage, Thermoscientific). All environmental data were deposited at the Knowledge Network for Biocomplexity (doi:10.5063/F1P26WH3).

### Biological components

The vegetation surrounding each ponds was assessed by determining the percent cover of each vascular plants and moss using a gridded quadrat of 50 × 50 cm^2^. Five quadrats were evaluated around each pond and the mean abundance of each species was used for subsequent analysis. Phytoplankton was collected in duplicates on glass fibre filters (Whatman GF/F, pore‐size: 0.7 μm), and stored frozen. Chl‐a was sequentially extracted with the following solvents: acetone: water (85:15 v/v), pure acetone and isopropanol: hexane (50:50 v/v). The extracts were vacuum concentrated, followed by volume reduction under a gentle stream of nitrogen, and stored at −80°C until their quantification with a U‐HPLC (Agilent 1290 Infinity) coupled with a diode array detector (Milani *et al*., 2019). Absorbance at 665 nm was used to measure Chl‐a concentration, identified with a standard (Sigma‐Aldrich). To estimate the abundance of the planktonic bacteria, unfiltered pond water was fixed with formaldehyde (2% v/v final concentration) and stored at 4°C for approximately 2–3 weeks before further analysis. Portions of 2 ml of the fixed water were filtered onto black polycarbonate filters (pore size: 0.22 μm) and stained with 4,6‐diamidino‐2‐phenylindole. A minimum of 800 cells per sample were counted under UV excitation by epifluorescence microscopy (Zeiss Axio Imager M1) at 1600× magnification (Posch *et al*., 2001).

In order to determine the composition of pond water microbial communities, 150 to 300 ml of pond surface water was filtered in duplicates onto 0.22 μm pore sized polyethersulfone filters (Millipore, GPWP). The filters were stored frozen until their analysis. The DNA was extracted with the DNeasy PowerBiofilm Kit (Qiagen, Germany) largely according to the manufacturer's specification, but with extending the inhibitor removal step to 1 h. The purified DNA was stored at −20°C in 10 mM Tris buffer. Partial ribosomal genes were amplified from the extracted DNA by PCR using the primer pair 799F‐1115R targeting bacteria with high specificity but excluding cyanobacteria and chloroplasts (Chelius and Triplett, 2001; Redford *et al*., 2010), and TAReuk454FWD1 – TAReukREV3 for eukaryotes (Stoeck *et al*., 2010). PCR products were sequenced on the Illumina MiSeq platform (2 x 300 bp, LGC genomics, Germany). Raw sequence data were deposited at the European Nucleotide Archive (PRJEB40506).

Raw data from rRNA gene sequencing were processed by an in‐house pipeline written in DELPHI, which included the joining of paired reads, quality control (0.5 average error rate per sequence), dereplication, exclusion of singleton reads, and a probabilistic clustering of operational taxonomic units (OTUs) comparable to UNOISE (Edgar and Flyvbjerg, 2015). Our algorithm first sorts sequences according to abundance, then assesses the probability that each queried sequence is derived from a more abundant ‘parent’, using published data to model the error probability of PCR amplification (propagating error) and sequencing (non‐propagating error). Then it assesses if the query sequence is chimeric using the whole data set (internal chimera check), and recursively assigns all sequences to their most likely ‘parents’. The remaining ‘ancestor’ sequences are retained as the most probable ‘true’ genotypes. Thus, there is no fixed distance threshold; instead, depending on the available information, sequence types with 2–3 mismatches can be discriminated. The pipeline was benchmarked using the Human Microbiome Mock Community (http://hmpdacc.org/HMMC/) against Uparse, Mothur, Qiime, Dada2 and Swarm, where it performed as well as Uparse in terms of accuracy.

The taxonomic assignment of OTUs was realized by a BLAST analysis of their representative sequences against the SILVA database (version 132) (Quast *et al*., 2012). Only bacterial OTUs with a query coverage ≥99% (i.e. 99% of bases from the OTU sequence could be aligned to a database sequence by BLAST) and eukaryotic OTUs with a query cover of 100% were included. Mitochondria and chloroplasts were removed from the bacterial OTU list, and spermatophytes and metazoans from the eukaryotic OTU list. A total of 3156 and 2968 OTUs were detected prior to normalization for bacteria and eukaryotes respectively. The OTU lists were deposited at the Knowledge Network for Biocomplexity (doi:10.5063/F1P26WH3).The two datasets were normalized to equal sample size using the ‘rarefy_even_depth’ function (R package ‘Phyloseq’) that randomly removes reads from the dataset using replacement (McMurdie and Holmes, 2013). This resulted in 2466 bacterial and 1591 eukaryotic OTUs with a sequencing depth of 7822 and 1460 reads per sample, respectively. The estimated abundances of methanotrophs were calculated from the proportions of their read numbers multiplied by total bacterial cell numbers as obtained from microscopic counts.

### Data analysis

All statistical analyses were carried out with the R software (version 3.6.1). Differences in environmental parameters between the two sets of ponds were tested by Mann–Whitney or *t*‐tests, depending on the normality and homoscedasticity of the data. False discovery rate of multiple testing was corrected using the Benjamini‐Hochberg method (Benjamini and Hochberg, 1995).

The *β*‐diversity of the communities was assessed using the BC similarity index, defined as 1 – BC dissimilarity with the ‘Vegan’ package (Oksanen *et al*., 2013). Duplicate microbial communities from single ponds were analysed separately, resulting in 20 data points per sites. BC similarity of bacterial and eukaryotic communities in such duplicate samples was high (mean BC similarity in polygonal ponds: bacteria 81%; eukaryotes 81%; in trough ponds: bacteria 71%; eukaryotes 76%). Moreover, samples taken from the same ponds were significantly more similar to each other than to samples taken from different ponds (Chi‐square tests: bacteria: *χ* ^2^ = 82, *p* < 0.01; eukaryotes: *χ* ^2^ = 115, *p* < 0.01).

Non‐metric multidimensional scaling was performed using ‘metaMDS’ and PERMANOVAs were computed with the ‘adonis’ function from the ‘Vegan’ package. The relative contribution of OTUs from different phylogenetic lineages to the average dissimilarity of polygonal and trough pond communities was assessed with the ‘simper’ function from the same package (Clarke, 1993). One replicate sample of pond B09 only contained a single eukaryotic read and was, therefore, excluded from the analysis. Similarity profile analysis (‘simprof’ with 999 permutations) from the ‘Clustsig’ package was used to assess the significance of dissimilarity between the samples (Clarke *et al*., 2008). The relationship between the collected environmental variables and the microbial communities was inferred by Mantel tests on the duplicates‐pooled datasets. A db‐RDA was computed on the pooled dataset with the function ‘capscale’ from the ‘vegan’ package to analyse which physico‐chemical parameters were the most important in shaping microbial community structure. The model was built on a reduced number of variables chosen with the function ‘bioenv’. Pond B05 was excluded due to its unique phytoplankton bloom.

In order to investigate the assembly processes of the microbial communities, the modified RC index that is based on read numbers (Chase *et al*., 2011) and the NST developed by Ning *et al*., (2019) were calculated using the ‘NST’ package. These indices use null model‐based approaches to quantify ecological stochasticity. The RC metric takes into account variability of α‐diversity; it indicates if two communities are more (−1 < RC < −0.95) or less (0.95 < RC < 1) similar than expected by chance or if stochastic processes dominate (−0.95 < RC < 0.95). Similarly, if NST < 50%, community assembly is predominantly governed by deterministic processes, while NST values >50% indicate the dominance of stochastic processes. For these analyses, the replicates from each pond were pooled. The NST index was also calculated separately for subcommunities of OTUS that were present in an increasing number of systems. This allows to assess the relative importance of stochastic and deterministic processes with respect to rarely occurring and more ubiquitous community members.

We used the βNTI to assess phylogenetic turnover across the ponds (Stegen *et al*., 2012; Stegen *et al*., 2013). For this purpose, the bacterial and eukaryotic OTUs were first independently aligned with PASTA using default settings (Mirarab *et al*., 2014), and maximum‐likelihood phylogenies were constructed using IQ‐TREE (Nguyen *et al*., 2015) with the best‐fitting substitution models chosen by ModelFinder (Kalyaanamoorthy *et al*., 2017) based on the Bayesian Information Criterion. The βNTIs were then calculated from phylogenetic distances using the code provided in (Stegen *et al*., 2013) with the ‘picante’ package. Communities are more closely related than expected by chance when βNTI >2, less closely related when βNTI < −2 and randomly related when −2 < βNTI < 2. To understand the impact of evaporation on the methane dynamics, correlation between δ^2^H‐H_2_O and methane concentration as well as δ^13^C‐CH_4_ was performed. The role of methanotrophs was assessed with correlations between the estimated abundance of methanotrophs and methane concentration as well as δ^13^C‐CH_4_. Additionally, methane concentrations and estimated abundance of methanotrophs were Box‐Cox transformed (Box and Cox, 1964) and a regression of methane concentration on estimated abundance of methanotrophs was calculated. Pond B05 was excluded from this analysis because there were no reads from methanotrophs in this system.

## Supporting information


**Fig. S1.** Orthomosaic of drone images of (A) site A, (B) site B. Points and label represent individual ponds sampled in this study.
**Fig. S2.** Hierarchical agglomerative clustering of the ponds based on the surrounding vegetation (distance: Bray‐Curtis, agglomeration: average). Colours represent significant clusters detected by similarity profile analysis (Simprof).
**Fig. S3.** Isotopic signature of pond water shown in relation to the Global Meteoric Water Line (GMWL).
**Fig. S4.** Phylogenetic relationship of methanotrophs (Maximum Likelihood method) and their relative abundance in each pond. Each circle represents one pond.
**Fig. S5.** db‐RDA of the (A) bacterial (B) micro‐eukaryotic communities. Shaded points represent the OTUs and arrows represent significant environmental parameters. On the axis, the percentage of inertia is represented for the fitted model and for the total inertia. Pond B05 was excluded from the analysis of the bacterial communities.
**Fig. S6.** A. Coefficient of variation of the physico‐chemical properties in pond B01 from the three sampling dates: t1: 13/07/2018, t2: 17/07/2018 and t3: 23/07/2018. Abbreviations are described in Table 1.
B. Bray‐Curtis dissimilarity for the prokaryotic and eukaryotic communities for the three sampling dates.
**Table S1.** Average read numbers of OTUs that significantly contributed to the difference of the bacterial microbial communities between polygonal and trough ponds (SIMPER analysis). Only OTUs with a contribution >1% are shown. Distance: percentage difference to closest know sequence (accession number).
**Table S2.** Average read numbers of OTUs that significantly contributed to the difference of the eukaryotic microbial communities between polygonal and trough ponds (SIMPER analysis). Only OTUs with a contribution >1% are shown. Distance: percentage difference to closest know sequence (accession number).

## References

[emi15260-bib-0001] Andresen, C.G. , and Lougheed, V.L. (2015) Disappearing Arctic tundra ponds: fine‐scale analysis of surface hydrology in drained thaw lake basins over a 65 year period (1948‐2013). J Geophys Res Biogeo 120: 466–479.

[emi15260-bib-0002] Bartosiewicz, M. , Laurion, I. , Clayer, F. , and Maranger, R. (2016) Heat‐wave effects on oxygen, nutrients, and phytoplankton can alter global warming potential of gases emitted from a small shallow lake. Environ Sci Technol 50: 6267–6275. 27266257 10.1021/acs.est.5b06312

[emi15260-bib-0003] Bartosiewicz, M. , Laurion, I. , and MacIntyre, S. (2015) Greenhouse gas emission and storage in a small shallow lake. Hydrobiologia 757: 101–115.

[emi15260-bib-0004] Bartosiewicz, M. , Przytulska, A. , Lapierre, J.F. , Laurion, I. , Lehmann, M.F. , and Maranger, R. (2019) Hot tops, cold bottoms: synergistic climate warming and shielding effects increase carbon burial in lakes. Limnol Oceanogr Lett 4: 132–144.

[emi15260-bib-0005] Benjamini, Y. , and Hochberg, Y. (1995) Controlling the false discovery rate: a practical and powerful approach to multiple testing. J R Stat Soc B Methodol 57: 289–300.

[emi15260-bib-0006] Blom, T. , Korhola, A. , Weckström, J. , Laing, T. , Snyder, J. , MacDonald, G. , and Smol, J. (2000) Physical and chemical characterisation of small subarctic headwater lakes in Finnish Lapland and the Kola Peninsula. Ver Int Ver Limnol 27: 316–320.

[emi15260-bib-0007] Bonilla, S. , Villeneuve, V. , and Vincent, W.F. (2005) Benthic and planktonic algal communities in a high Arctic Lake: pigment structure and contrasting responses to nutrient enrichment. J Phycol 41: 1120–1130.

[emi15260-bib-0008] Box, G.E. , and Cox, D.R. (1964) An analysis of transformations. J R Stat Soc B Methodol 26: 211–243.

[emi15260-bib-0009] Breton, J. , Prairie, Y. , Vallières, C. , and Laurion, I. (2009) Limnological properties of permafrost thaw ponds in northeastern Canada. Can J Fish Aquat Sci 66: 1635–1648.

[emi15260-bib-0010] Chase, J.M. , Kraft, N.J.B. , Smith, K.G. , Vellend, M. , and Inouye, B.D. (2011) Using null models to disentangle variation in community dissimilarity from variation in α‐diversity. Ecosphere 2: art24.

[emi15260-bib-0011] Chelius, M.K. , and Triplett, E.W. (2001) The diversity of archaea and bacteria in association with the roots of *Zea mays* L. Microb Ecol 41: 252–263. 11391463 10.1007/s002480000087

[emi15260-bib-0012] Clarke, K.R. (1993) Non‐parametric multivariate analyses of changes in community structure. Austral Ecol 18: 117–143.

[emi15260-bib-0013] Clarke, K.R. , Somerfield, P.J. , and Gorley, R.N. (2008) Testing of null hypotheses in exploratory community analyses: similarity profiles and biota‐environment linkage. J Exp Mar Biol Ecol 366: 56–69.

[emi15260-bib-0014] Comte, J. , Monier, A. , Crevecoeur, S. , Lovejoy, C. , and Vincent, W.F. (2016) Microbial biogeography of permafrost thaw ponds across the changing northern landscape. Ecography 39: 609–618.

[emi15260-bib-0015] Crevecoeur, S. , Vincent, W.F. , Comte, J. , and Lovejoy, C. (2015) Bacterial community structure across environmental gradients in permafrost thaw ponds: methanotroph‐rich ecosystems. Front Microbiol 6: 192. 25926816 10.3389/fmicb.2015.00192 PMC4396522

[emi15260-bib-0016] Crevecoeur, S. , Vincent, W.F. , Comte, J. , Matveev, A. , and Lovejoy, C. (2017) Diversity and potential activity of methanotrophs in high methane‐emitting permafrost thaw ponds. PLoS One 12: e0188223. 29182670 10.1371/journal.pone.0188223 PMC5705078

[emi15260-bib-0017] Crump, B.C. , Amaral‐Zettler, L.A. , and Kling, G.W. (2012) Microbial diversity in arctic freshwaters is structured by inoculation of microbes from soils. ISME J 6: 1629–1639. 22378536 10.1038/ismej.2012.9 PMC3498914

[emi15260-bib-0018] Crump, B.C. , Kling, G.W. , Bahr, M. , and Hobbie, J.E. (2003) Bacterioplankton community shifts in an arctic lake correlate with seasonal changes in organic matter source. Appl Environ Microbiol 69: 2253–2268. 12676708 10.1128/AEM.69.4.2253-2268.2003 PMC154827

[emi15260-bib-0019] Dedysh, S.N. (2009) Exploring methanotroph diversity in acidic northern wetlands: molecular and cultivation‐based studies. Microbiology 78: 655–669.

[emi15260-bib-0020] Del Giorgio, P.A. , and Cole, J.J. (1998) Bacterial growth efficiency in natural aquatic systems. Annu Rev Ecol Syst 29: 503–541.

[emi15260-bib-0021] Deshpande, B.N. , Crevecoeur, S. , Matveev, A. , and Vincent, W.F. (2016) Bacterial production in subarctic peatland lakes enriched by thawing permafrost. Biogeosciences 13: 4411–4427.

[emi15260-bib-0022] Dinsmore, K.J. , Billett, M.F. , and Dyson, K.E. (2013) Temperature and precipitation drive temporal variability in aquatic carbon and GHG concentrations and fluxes in a peatland catchment. Glob Chang Biol 19: 2133–2148. 23568485 10.1111/gcb.12209

[emi15260-bib-0023] Edgar, R.C. , and Flyvbjerg, H. (2015) Error filtering, pair assembly and error correction for next‐generation sequencing reads. Bioinformatics 31: 3476–3482. 26139637 10.1093/bioinformatics/btv401

[emi15260-bib-0024] Fasching, C. , Behounek, B. , Singer, G.A. , and Battin, T.J. (2014) Microbial degradation of terrigenous dissolved organic matter and potential consequences for carbon cycling in brown‐water streams. Sci Rep 4: 4981. 24828296 10.1038/srep04981 PMC4021337

[emi15260-bib-0025] Fukami, T. (2015) Historical contingency in community assembly: integrating niches, species pools, and priority effects. Annu Rev Ecol Evol Syst 46: 1–23.

[emi15260-bib-0026] Graef, C. , Hestnes, A.G. , Svenning, M.M. , and Frenzel, P. (2011) The active methanotrophic community in a wetland from the High Arctic. Environ Microbiol Rep 3: 466–472. 23761309 10.1111/j.1758-2229.2010.00237.x

[emi15260-bib-0027] Graham, E.B. , Knelman, J.E. , Schindlbacher, A. , Siciliano, S. , Breulmann, M. , Yannarell, A. , *et al* (2016) Microbes as engines of ecosystem function: when does community structure enhance predictions of ecosystem processes? Front Microbiol 7: 214. 26941732 10.3389/fmicb.2016.00214 PMC4764795

[emi15260-bib-0028] Green, J.L. , Holmes, A.J. , Westoby, M. , Oliver, I. , Briscoe, D. , Dangerfield, M. , *et al* (2004) Spatial scaling of microbial eukaryote diversity. Nature 432: 747–750. 15592411 10.1038/nature03034

[emi15260-bib-0029] Grosse, G. , Robinson, J.E. , Bryant, R. , Taylor, M.D. , Harper, W. , DeMasi, A. , *et al* (2013) Distribution of late Pleistocene ice‐rich syngenetic permafrost of the Yedoma Suite in east and central Siberia, Russia. U.S.G.S. Open File Report 2013: 1–37.

[emi15260-bib-0030] Hahn, M.W. (2003) Isolation of strains belonging to the cosmopolitan Polynucleobacter necessarius cluster from freshwater habitats located in three climatic zones. Appl Environ Microbiol 69: 5248–5254. 12957910 10.1128/AEM.69.9.5248-5254.2003 PMC194981

[emi15260-bib-0031] He, R. , Wooller, M.J. , Pohlman, J.W. , Quensen, J. , Tiedje, J.M. , and Leigh, M.B. (2012) Diversity of active aerobic methanotrophs along depth profiles of arctic and subarctic lake water column and sediments. ISME J 6: 1937–1948. 22592821 10.1038/ismej.2012.34 PMC3446799

[emi15260-bib-0032] Helms, J.R. , Stubbins, A. , Ritchie, J.D. , Minor, E.C. , Kieber, D.J. , and Mopper, K. (2008) Absorption spectral slopes and slope ratios as indicators of molecular weight, source, and photobleaching of chromophoric dissolved organic matter. Limnol Oceanogr 53: 955–969.

[emi15260-bib-0102] Juszak, I. , Eugster, W. , Heijmans, M.M.P.D. , and Schaepman‐Strub, G. (2016). Contrasting radiation and soil heat fluxes in Arctic shrub and wet sedge tundra. Biogeosciences 13:4049–4064.

[emi15260-bib-0033] Kalyaanamoorthy, S. , Minh, B.Q. , Wong, T.K.F. , von Haeseler, A. , and Jermiin, L.S. (2017) ModelFinder: fast model selection for accurate phylogenetic estimates. Nat Methods 14: 587–589. 28481363 10.1038/nmeth.4285 PMC5453245

[emi15260-bib-0034] Kling, G.W. , Kipphut, G.W. , and Miller, M.C. (1992) The flux of CO_2_ and CH_4_ from lakes and rivers in arctic Alaska. Hydrobiologia 240: 23–36.

[emi15260-bib-0035] Laas, A. , Nõges, P. , Kõiv, T. , and Nõges, T. (2012) High‐frequency metabolism study in a large and shallow temperate lake reveals seasonal switching between net autotrophy and net heterotrophy. Hydrobiologia 694: 57–74.

[emi15260-bib-0036] Lachenbruch, A.H. (1962) *Mechanics of thermal contraction cracks and ice‐wedge polygons in permafrost* . Geological Society of America. Special Papers No. 70, 1962, vii, 69, p., illus

[emi15260-bib-0037] Langenheder, S. , Lindstrom, E.S. , and Tranvik, L.J. (2006) Structure and function of bacterial communities emerging from different sources under identical conditions. Appl Environ Microbiol 72: 212–220. 16391045 10.1128/AEM.72.1.212-220.2006 PMC1352196

[emi15260-bib-0038] Langenheder, S. , Wang, J. , Karjalainen, S.M. , Laamanen, T.M. , Tolonen, K.T. , Vilmi, A. , and Heino, J. (2017) Bacterial metacommunity organization in a highly connected aquatic system. FEMS Microbiol Ecol 93 10.1093/femsec/fiw225. 27810879

[emi15260-bib-0039] Larouche, J.R. , Abbott, B.W. , Bowden, W.B. , and Jones, J.B. (2015) The role of watershed characteristics, permafrost thaw, and wildfire on dissolved organic carbon biodegradability and water chemistry in Arctic headwater streams. Biogeosciences 12: 4021–4056.

[emi15260-bib-0040] Laurion, I. , and Mladenov, N. (2013) Dissolved organic matter photolysis in Canadian arctic thaw ponds. Environ Res Lett 8: 35026.

[emi15260-bib-0041] Laurion, I. , Vincent, W.F. , MacIntyre, S. , Retamal, L. , Dupont, C. , Francus, P. , and Pienitz, R. (2010) Variability in greenhouse gas emissions from permafrost thaw ponds. Limnol Oceanogr 55: 115–133.

[emi15260-bib-0042] Li, B. , Heijmans, M.M.P.D. , Blok, D. , Wang, P. , Karsanaev, S.V. , Maximov, T.C. , *et al* (2017) Thaw pond development and initial vegetation succession in experimental plots at a Siberian lowland tundra site. Plant and Soil 420: 147–162.

[emi15260-bib-0043] Liu, L. , Yang, J. , Yu, Z. , and Wilkinson, D.M. (2015) The biogeography of abundant and rare bacterioplankton in the lakes and reservoirs of China. ISME J 9: 2068–2077. 25748371 10.1038/ismej.2015.29 PMC4542038

[emi15260-bib-0044] Logue, J.B. , Langenheder, S. , Andersson, A.F. , Bertilsson, S. , Drakare, S. , Lanzen, A. , and Lindstrom, E.S. (2012) Freshwater bacterioplankton richness in oligotrophic lakes depends on nutrient availability rather than on species‐area relationships. ISME J 6: 1127–1136. 22170419 10.1038/ismej.2011.184 PMC3358030

[emi15260-bib-0045] Lüke, C. , and Frenzel, P. (2011) Potential of pmoA amplicon pyrosequencing for methanotroph diversity studies. Appl Environ Microbiol 77: 6305–6309. 21764977 10.1128/AEM.05355-11 PMC3165393

[emi15260-bib-0046] Martiny, J.B. , Eisen, J.A. , Penn, K. , Allison, S.D. , and Horner‐Devine, M.C. (2011) Drivers of bacterial beta‐diversity depend on spatial scale. Proc Natl Acad Sci U S A 108: 7850–7854. 21518859 10.1073/pnas.1016308108 PMC3093525

[emi15260-bib-0047] McMurdie, P.J. , and Holmes, S. (2013) phyloseq: an R package for reproducible interactive analysis and graphics of microbiome census data. PLoS One 8: e61217. 23630581 10.1371/journal.pone.0061217 PMC3632530

[emi15260-bib-0048] Menezes, M. , and Novarino, G. (2003) How diverse are planktonic cryptomonads in Brazil? Advantages and difficulties of a taxonomic‐biogeographical approach. Hydrobiologia 502: 297–306.

[emi15260-bib-0049] Milani, G. , Kneubühler, M. , Tonolla, D. , Doering, M. , Wiesenberg, G. , and Schaepman, M. (2019) Remotely sensing variation in ecological strategies and plant traits of willows in perialpine floodplains. J Geophys Res Biogeo 124: 2090–2106.

[emi15260-bib-0050] Mirarab, S. , Nguyen, N. , and Warnow, T. (2014) PASTA: ultra‐large multiple sequence alignment In International Conference on Research in Computational Molecular Biology, Cham, Switzerland: Springer, pp. 177–191.

[emi15260-bib-0051] Muscarella, M.E. , Boot, C.M. , Broeckling, C.D. , and Lennon, J.T. (2019) Resource heterogeneity structures aquatic bacterial communities. ISME J 13: 2183–2195. 31053829 10.1038/s41396-019-0427-7 PMC6775984

[emi15260-bib-0052] Muster, S. , Langer, M. , Heim, B. , Westermann, S. , and Boike, J. (2012) Subpixel heterogeneity of ice‐wedge polygonal tundra: a multi‐scale analysis of land cover and evapotranspiration in the Lena River Delta, Siberia. Tellus B Chem Phys Meteorol 64: 17301–17320. 10.3402/tellusb.v64i0.17301.

[emi15260-bib-0053] Muster, S. , Roth, K. , Langer, M. , Lange, S. , Cresto Aleina, F. , Bartsch, A. , *et al* (2017) PeRL: a circum‐Arctic Permafrost Region Pond and Lake database. Earth Syst Sci Data 9: 317–348.

[emi15260-bib-0054] Negandhi, K. , Laurion, I. , and Lovejoy, C. (2014) Bacterial communities and greenhouse gas emissions of shallow ponds in the High Arctic. Polar Biol 37: 1669–1683.

[emi15260-bib-0055] Negandhi, K. , Laurion, I. , and Lovejoy, C. (2016) Temperature effects on net greenhouse gas production and bacterial communities in arctic thaw ponds. FEMS Microbiol Ecol 92: fiw117. 27288196 10.1093/femsec/fiw117

[emi15260-bib-0056] Nemergut, D.R. , Schmidt, S.K. , Fukami, T. , O'Neill, S.P. , Bilinski, T.M. , Stanish, L.F. , *et al* (2013) Patterns and processes of microbial community assembly. Microbiol Mol Biol Rev 77: 342–356. 24006468 10.1128/MMBR.00051-12 PMC3811611

[emi15260-bib-0057] Nguyen, L.T. , Schmidt, H.A. , von Haeseler, A. , and Minh, B.Q. (2015) IQ‐TREE: a fast and effective stochastic algorithm for estimating maximum‐likelihood phylogenies. Mol Biol Evol 32: 268–274. 25371430 10.1093/molbev/msu300 PMC4271533

[emi15260-bib-0058] Ning, D. , Deng, Y. , Tiedje, J.M. , and Zhou, J. (2019) A general framework for quantitatively assessing ecological stochasticity. Proc Natl Acad Sci U S A 116: 16892–16898. 31391302 10.1073/pnas.1904623116 PMC6708315

[emi15260-bib-0059] Nino‐Garcia, J.P. , Ruiz‐Gonzalez, C. , and Del Giorgio, P.A. (2016) Landscape‐scale spatial abundance distributions discriminate core from random components of boreal lake bacterioplankton. Ecol Lett 19: 1506–1515. 27882701 10.1111/ele.12704

[emi15260-bib-0060] Oksanen, J. , Blanchet, F.G. , Kindt, R. , Legendre, P. , Minchin, P.R. , O'hara, R. , *et al* (2013) Vegan: Community Ecology Package. R Package Version. 2.0‐10. CRAN, 1–295.

[emi15260-bib-0061] Ostman, O. , Drakare, S. , Kritzberg, E.S. , Langenheder, S. , Logue, J.B. , and Lindstrom, E.S. (2010) Regional invariance among microbial communities. Ecol Lett 13: 118–127. 19968693 10.1111/j.1461-0248.2009.01413.x

[emi15260-bib-0062] Osudar, R. , Liebner, S. , Alawi, M. , Yang, S. , Bussmann, I. , and Wagner, D. (2016) Methane turnover and methanotrophic communities in arctic aquatic ecosystems of the Lena Delta, Northeast Siberia. FEMS Microbiol Ecol 92: fiw116. 27230921 10.1093/femsec/fiw116

[emi15260-bib-0063] Payette, S. (2004) Accelerated thawing of subarctic peatland permafrost over the last 50 years. Geophys Res Lett 31 10.1029/2004GL020358.

[emi15260-bib-0064] Peura, S. , Wauthy, M. , Simone, D. , Eiler, A. , Einarsdóttir, K. , Rautio, M. , and Bertilsson, S. (2020) Ontogenic succession of thermokarst thaw ponds is linked to dissolved organic matter quality and microbial degradation potential. Limnol Oceanogr 65: S248–S263.

[emi15260-bib-0065] Pienitz, R. , Doran, P.T. , and Lamoureux, S.F. (2008) Origin and geomorphology of lakes in the polar regions In Polar Lakes and Rivers: Limnology of Arctic and Antarctic Aquatic Ecosystems, (pp. 25–41). Oxford, England: Oxford University Press.

[emi15260-bib-0066] Pierre, K.A. , Louis, V.L. , Schiff, S.L. , Lehnherr, I. , Dainard, P.G. , Gardner, A.S. , *et al* (2019) Proglacial freshwaters are significant and previously unrecognized sinks of atmospheric CO2. Proc Natl Acad Sci U S A 116: 17690–17695. 31427515 10.1073/pnas.1904241116 PMC6731667

[emi15260-bib-0067] Pokrovsky, O.S. , Shirokova, L.S. , Kirpotin, S.N. , Audry, S. , Viers, J. , and Dupré, B. (2011) Effect of permafrost thawing on organic carbon and trace element colloidal speciation in the thermokarst lakes of western Siberia. Biogeosciences 8: 565–583.

[emi15260-bib-0068] Posch, T. , Loferer‐Krößbacher, M. , Gao, G. , Alfreider, A. , Pernthaler, J. , and Psenner, R. (2001) Precision of bacterioplankton biomass determination: a comparison of two fluorescent dyes, and of allometric and linear volume‐to‐carbon conversion factors. Aquat Microb Ecol 25: 55–63.

[emi15260-bib-0069] Przytulska, A. , Comte, J. , Crevecoeur, S. , Lovejoy, C. , Laurion, I. , and Vincent, W.F. (2016) Phototrophic pigment diversity and picophytoplankton in permafrost thaw lakes. Biogeosciences 13: 13–26.

[emi15260-bib-0070] Quast, C. , Pruesse, E. , Yilmaz, P. , Gerken, J. , Schweer, T. , Yarza, P. , *et al* (2012) The SILVA ribosomal RNA gene database project: improved data processing and web‐based tools. Nucleic Acids Res 41: D590–D596. 23193283 10.1093/nar/gks1219 PMC3531112

[emi15260-bib-0071] Rautio, M. , Dufresne, F. , Laurion, I. , Bonilla, S. , Vincent, W.F. , and Christoffersen, K.S. (2011) Shallow freshwater ecosystems of the circumpolar Arctic. Écoscience 18: 204–222.

[emi15260-bib-0072] Redford, A.J. , Bowers, R.M. , Knight, R. , Linhart, Y. , and Fierer, N. (2010) The ecology of the phyllosphere: geographic and phylogenetic variability in the distribution of bacteria on tree leaves. Environ Microbiol 12: 2885–2893. 20545741 10.1111/j.1462-2920.2010.02258.x PMC3156554

[emi15260-bib-0073] Repo, E. , Huttunen, J.T. , Naumov, A.V. , Chichulin, A.V. , Lapshina, E.D. , Bleuten, W. , and Martikainen, P.J. (2007) Release of CO2 and CH4 from small wetland lakes in western Siberia. Tellus B Chem Phys Meteorol 59: 788–796.

[emi15260-bib-0074] Riordan, B. , Verbyla, D. , and McGuire, A.D. (2006) Shrinking ponds in subarctic Alaska based on 1950–2002 remotely sensed images. J Geophys Res Biogeosci 111 10.1029/2005JG000150.

[emi15260-bib-0075] Roiha, T. , Tiirola, M. , Cazzanelli, M. , and Rautio, M. (2012) Carbon quantity defines productivity while its quality defines community composition of bacterioplankton in subarctic ponds. Aquat Sci 74: 513–525.

[emi15260-bib-0076] Saad, J.F. , Schiaffino, M.R. , Vinocur, A. , O'Farrell, I. , Tell, G. , and Izaguirre, I. (2013) Microbial planktonic communities of freshwater environments from Tierra del Fuego: dominant trophic strategies in lakes with contrasting features. J Plankton Res 35: 1220–1233.

[emi15260-bib-0077] Sarnelle, O. (2005) Daphnia as keystone predators: effects on phytoplankton diversity and grazing resistance. J Plankton Res 27: 1229–1238.

[emi15260-bib-0078] Schindler, D.E. , Carpenter, S.R. , Cole, J.J. , Kitchell, J.F. , and Pace, M.L. (1997) Influence of food web structure on carbon exchange between lakes and the atmosphere. Science 277: 248–251.

[emi15260-bib-0079] Schuur, E.A. , McGuire, A.D. , Schadel, C. , Grosse, G. , Harden, J.W. , Hayes, D.J. , *et al* (2015) Climate change and the permafrost carbon feedback. Nature 520: 171–179. 25855454 10.1038/nature14338

[emi15260-bib-0080] Shirokova, L.S. , Pokrovsky, O.S. , Kirpotin, S.N. , and Dupré, B. (2009) Heterotrophic bacterio‐plankton in thawed lakes of the northern part of Western Siberia controls the CO_2_ flux to the atmosphere. Int J Environ Stud 66: 433–445.

[emi15260-bib-0081] Smith, R.L. , Howes, B. , and Garabedian, S. (1991) In situ measurement of methane oxidation in groundwater by using natural‐gradient tracer tests. Appl Environ Microbiol 57: 1997–2004. 1892389 10.1128/aem.57.7.1997-2004.1991 PMC183511

[emi15260-bib-0082] Sobek, S. , Tranvik, L.J. , and Cole, J.J. (2005) Temperature independence of carbon dioxide supersaturation in global lakes. Global Biogeochem Cycles 19 10.1029/2004GB002264.

[emi15260-bib-0083] Stegen, J.C. , Lin, X. , Fredrickson, J.K. , Chen, X. , Kennedy, D.W. , Murray, C.J. , *et al* (2013) Quantifying community assembly processes and identifying features that impose them. ISME J 7: 2069–2079. 23739053 10.1038/ismej.2013.93 PMC3806266

[emi15260-bib-0084] Stegen, J.C. , Lin, X. , Konopka, A.E. , and Fredrickson, J.K. (2012) Stochastic and deterministic assembly processes in subsurface microbial communities. ISME J 6: 1653–1664. 22456445 10.1038/ismej.2012.22 PMC3498916

[emi15260-bib-0085] Stoeck, T. , Bass, D. , Nebel, M. , Christen, R. , Jones, M.D. , Breiner, H.W. , and Richards, T.A. (2010) Multiple marker parallel tag environmental DNA sequencing reveals a highly complex eukaryotic community in marine anoxic water. Mol Ecol 19 (Suppl 1): 21–31. 20331767 10.1111/j.1365-294X.2009.04480.x

[emi15260-bib-0086] Strauss, J. , Schirrmeister, L. , Grosse, G. , Wetterich, S. , Ulrich, M. , Herzschuh, U. , and Hubberten, H.W. (2013) The deep permafrost carbon pool of the Yedoma region in Siberia and Alaska. Geophys Res Lett 40: 6165–6170. 26074633 10.1002/2013GL058088 PMC4459201

[emi15260-bib-0087] Tarnocai, C. , Canadell, J.G. , Schuur, E.A.G. , Kuhry, P. , Mazhitova, G. , and Zimov, S. (2009) Soil organic carbon pools in the northern circumpolar permafrost region. Global Biogeochem Cycles 23 10.1029/2008GB003327.

[emi15260-bib-0088] Thrane, J.‐E. , Hessen, D.O. , and Andersen, T. (2014) The absorption of light in lakes: negative impact of dissolved organic carbon on primary productivity. Ecosystems 17: 1040–1052.

[emi15260-bib-0089] Urban, M.C. (2004) Disturbance heterogeneity determines freshwater metacommunity structure. Ecology 85: 2971–2978.

[emi15260-bib-0090] Vähätalo, A.V. , Salonen, K. , Münster, U. , Järvinen, M. , and Wetzel, R.G. (2003) Photochemical transformation of allochthonous organic matter provides bioavailable nutrients in a humic lake. Archiv Fur Hydrobiologie 156: 287–314.

[emi15260-bib-0091] van Huissteden, J. , Berrittella, C. , Parmentier, F.J.W. , Mi, Y. , Maximov, T.C. , and Dolman, A.J. (2011) Methane emissions from permafrost thaw lakes limited by lake drainage. Nat Clim Change 1: 119–123.

[emi15260-bib-0092] Verpoorter, C. , Kutser, T. , Seekell, D.A. , and Tranvik, L.J. (2014) A global inventory of lakes based on high‐resolution satellite imagery. Geophys Res Lett 41: 6396–6402.

[emi15260-bib-0093] Vincent, W.F. , Laurion, I. , Pienitz, R. , and Walter Anthony, K. (2013) Climate impacts on Arctic lake ecosystems In Climatic Change and Global Warming of Inland Waters: Impacts and Mitigation for Ecosystems and Societies, (pp. 27–42). Oxford, England: John Wiley & Sons, Ltd.

[emi15260-bib-0094] Vonk, J.E. , Tank, S.E. , Bowden, W.B. , Laurion, I. , Vincent, W.F. , Alekseychik, P. , *et al* (2015) Reviews and syntheses: effects of permafrost thaw on Arctic aquatic ecosystems. Biogeosciences 12: 7129–7167.

[emi15260-bib-0095] Walter, K.M. , Zimov, S.A. , Chanton, J.P. , Verbyla, D. , and Chapin, F.S., 3rd . (2006) Methane bubbling from Siberian thaw lakes as a positive feedback to climate warming. Nature 443: 71–75. 16957728 10.1038/nature05040

[emi15260-bib-0096] Wauthy, M. , Rautio, M. , Christoffersen, K.S. , Forsström, L. , Laurion, I. , Mariash, H.L. , *et al* (2018) Increasing dominance of terrigenous organic matter in circumpolar freshwaters due to permafrost thaw. Limnol Oceanogr Lett 3: 186–198.

[emi15260-bib-0097] Weishaar, J.L. , Aiken, G.R. , Bergamaschi, B.A. , Fram, M.S. , Fujii, R. , and Mopper, K. (2003) Evaluation of specific ultraviolet absorbance as an indicator of the chemical composition and reactivity of dissolved organic carbon. Environ Sci Technol 37: 4702–4708. 14594381 10.1021/es030360x

[emi15260-bib-0098] Wik, M. , Varner, R.K. , Anthony, K.W. , MacIntyre, S. , and Bastviken, D. (2016) Climate‐sensitive northern lakes and ponds are critical components of methane release. Nat Geosci 9: 99–105.

[emi15260-bib-0099] Yvon‐Durocher, G. , Allen, A.P. , Bastviken, D. , Conrad, R. , Gudasz, C. , St‐Pierre, A. , *et al* (2014) Methane fluxes show consistent temperature dependence across microbial to ecosystem scales. Nature 507: 488–491. 24670769 10.1038/nature13164

[emi15260-bib-0100] Zhou, J. , and Ning, D. (2017) Stochastic community assembly: does it matter in microbial ecology? Microbiol Mol Biol Rev 81: e00002–e00017. 29021219 10.1128/MMBR.00002-17 PMC5706748

[emi15260-bib-0101] Zöllner, E. , Santer, B. , Boersma, M. , Hoppe, H.G. , and Jürgens, K. (2003) Cascading predation effects of Daphnia and copepods on microbial food web components. Freshw Biol 48: 2174–2193.

