# Supplementary material for: The biogeochemical variability of Arctic thermokarst ponds is reflected by stochastic and niche‐driven microbial community assembly processes
Source: Environ Microbiol. 2020 Oct 13;22(11):4847–62. doi: 10.1111/1462-2920.15260 (PMC7702111; doi:10.1111/1462-2920.15260)
Supplement: Supplementary file 1 — Fig. S1. Orthomosaic of drone images of (A) site A, (B) site B. Points and label represent individual ponds sampled in this study. Fig. S2. Hierarchical agglomerative clustering of the ponds based on the surrounding vegetation (distance: Bray‐Curtis, agglomeration: average). Colours represent significant clusters detected by similarity profile analysis (Simprof). Fig. S3. Isotopic signature of pond water shown in relation to the Global Meteoric Water Line (GMWL). Fig. S4. Phylogenetic relationship of methanotrophs (Maximum Likelihood method) and their relative abundance in each pond. Each circle represents one pond. Fig. S5. db‐RDA of the (A) bacterial (B) micro‐eukaryotic communities. Shaded points represent the OTUs and arrows represent significant environmental parameters. On the axis, the percentage of inertia is represented for the fitted model and for the total inertia. Pond B05 was excluded from the analysis of the bacterial communities. Fig. S6. A. Coefficient of variation of the physico‐chemical properties in pond B01 from the three sampling dates: t1: 13/07/2018, t2: 17/07/2018 and t3: 23/07/2018. Abbreviations are described in Table 1. B. Bray‐Curtis dissimilarity for the prokaryotic and eukaryotic communities for the three sampling dates. Table S1. Average read numbers of OTUs that significantly contributed to the difference of the bacterial microbial communities between polygonal and trough ponds (SIMPER analysis). Only OTUs with a contribution >1% are shown. Distance: percentage difference to closest know sequence (accession number). Table S2. Average read numbers of OTUs that significantly contributed to the difference of the eukaryotic microbial communities between polygonal and trough ponds (SIMPER analysis). Only OTUs with a contribution >1% are shown. Distance: percentage difference to closest know sequence (accession number). [file EMI-22-4847-s001.pdf]

Supporting Information

a)

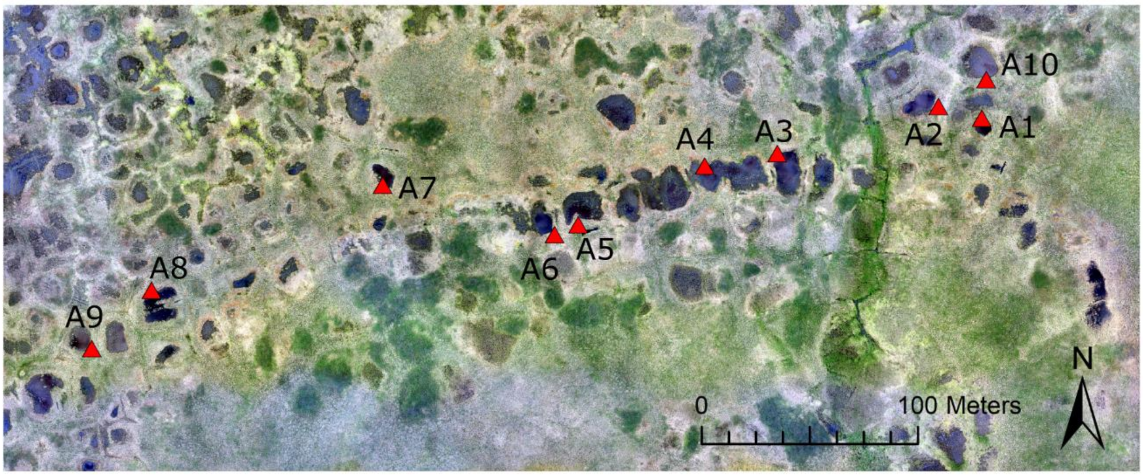

b)

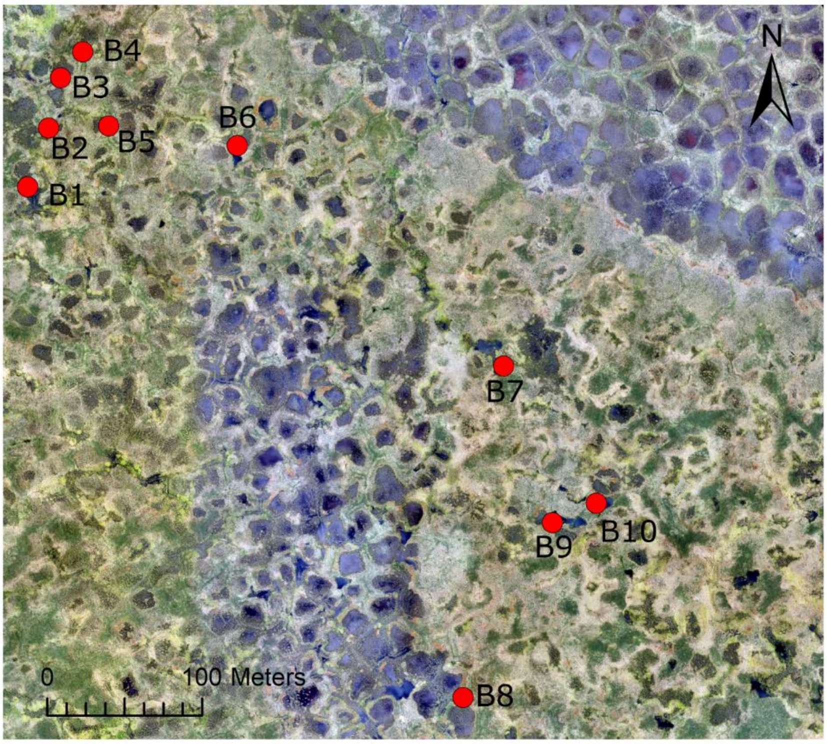

Figure S1: Orthomosaic of drone images of a) Site A, b) Site B. Points and label represent individual ponds sampled in this study.

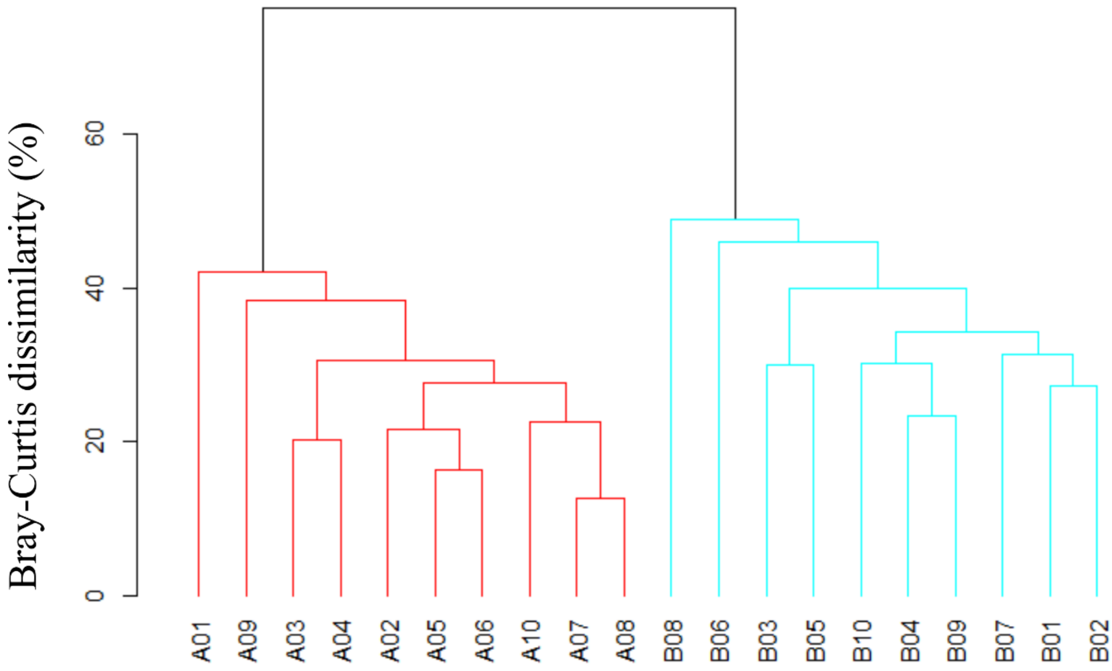

Figure S2: Hierarchical agglomerative clustering of the ponds based on the surrounding vegetation (Distance: Bray-Curtis, Agglomeration: average). Colours represent significant clusters detected by similarity profile analysis (Simprof).

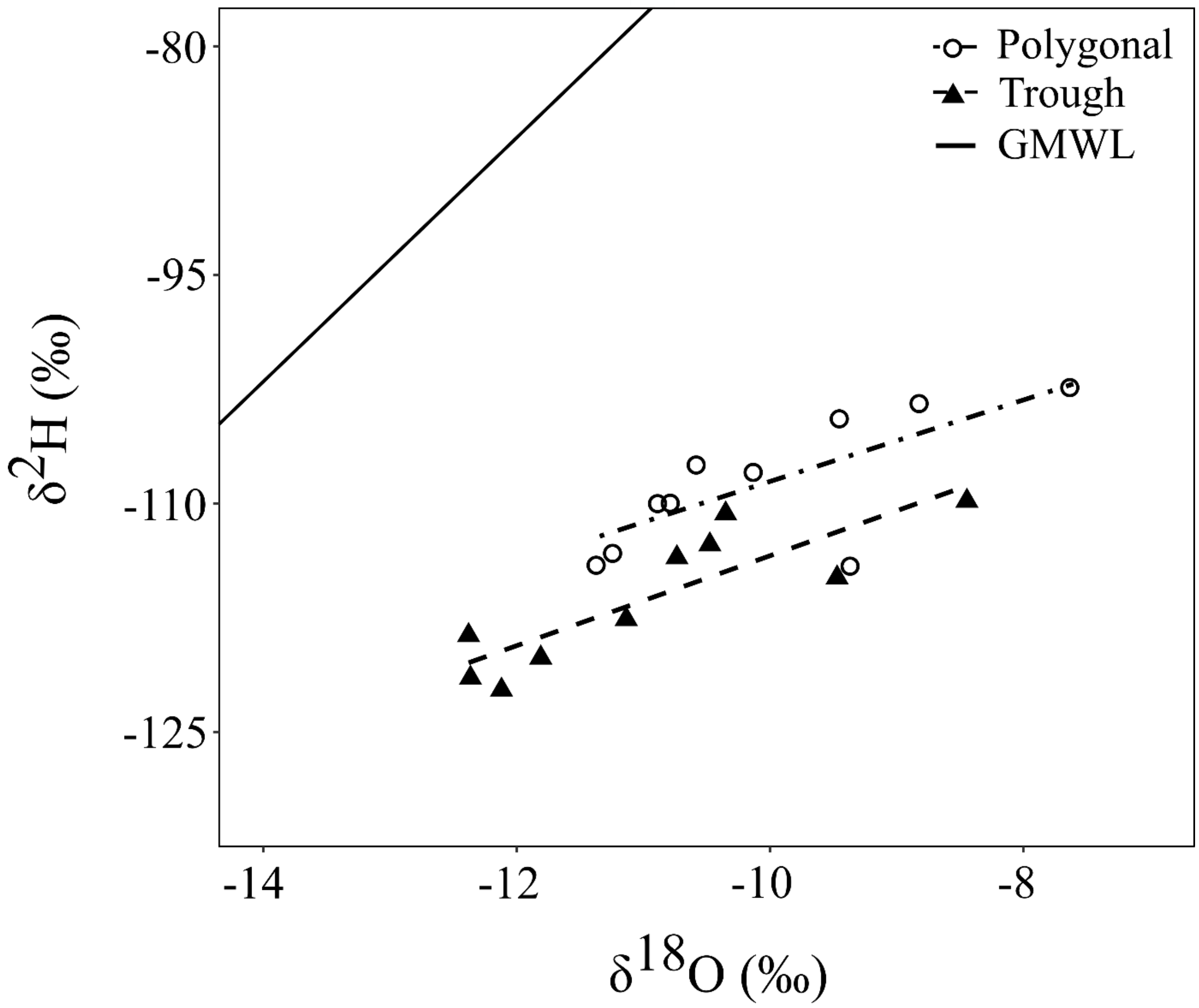

Figure S3: Isotopic signature of pond water shown in relation to the Global Meteoric Water Line (GMWL).

Table S1: Average read numbers of OTUs that significantly contributed to the difference of the bacterial microbial communities between polygonal and trough ponds (simper analysis). Only OTUs with a contribution >1 % are shown. Distance: % difference to closest know sequence (accession number).

OTUs	Polygonal	Trough	% contribution	p-value	Accession Number	Distance (%)	Phylum	Genus
OTU 2	532.6	1505	10.36	0.003	MT102304	0	Proteobacteria	*Variovorax/Rhodoferax1*
OTU 4	45.5	658.75	5.40	0.001	AM939566	0.7	Actinobacteria	*Candidatus Planktoluna*
OTU 5	596.15	9.35	5.01	0.001	FPLS01032293	0	Proteobacteria	*Alcaligenes*
OTU 7	105.1	542.25	4.36	0.007	EU801768	0	Proteobacteria	*Limnohabitans*
OTU 6	32.5	471.35	3.85	0.001	EU801564	0	Proteobacteria	*Limnohabitans*
OTU 10	441.45	2.25	3.73	0.001	EU800868	0	Actinobacteria	*Rhodoluna*
OTU 9	315.4	1	2.67	0.001	JN626726	0	Actinobacteria	*Nanopelagicales*
OTU 12	6.6	278.7	2.31	0.001	HM319621	0	Proteobacteria	*Rhodoblastus*
OTU 13	220.85	1.2	1.86	0.001	AJ290032	0	Proteobacteria	*Methylorosula*
OTU 11	204.95	75.45	1.61	0.036	JN656918	0	Actinobacteria	*Rhodoluna*
OTU 16	132.5	0	1.12	0.001	CP016780	0	Actinobacteria	*Nanopelagicales*

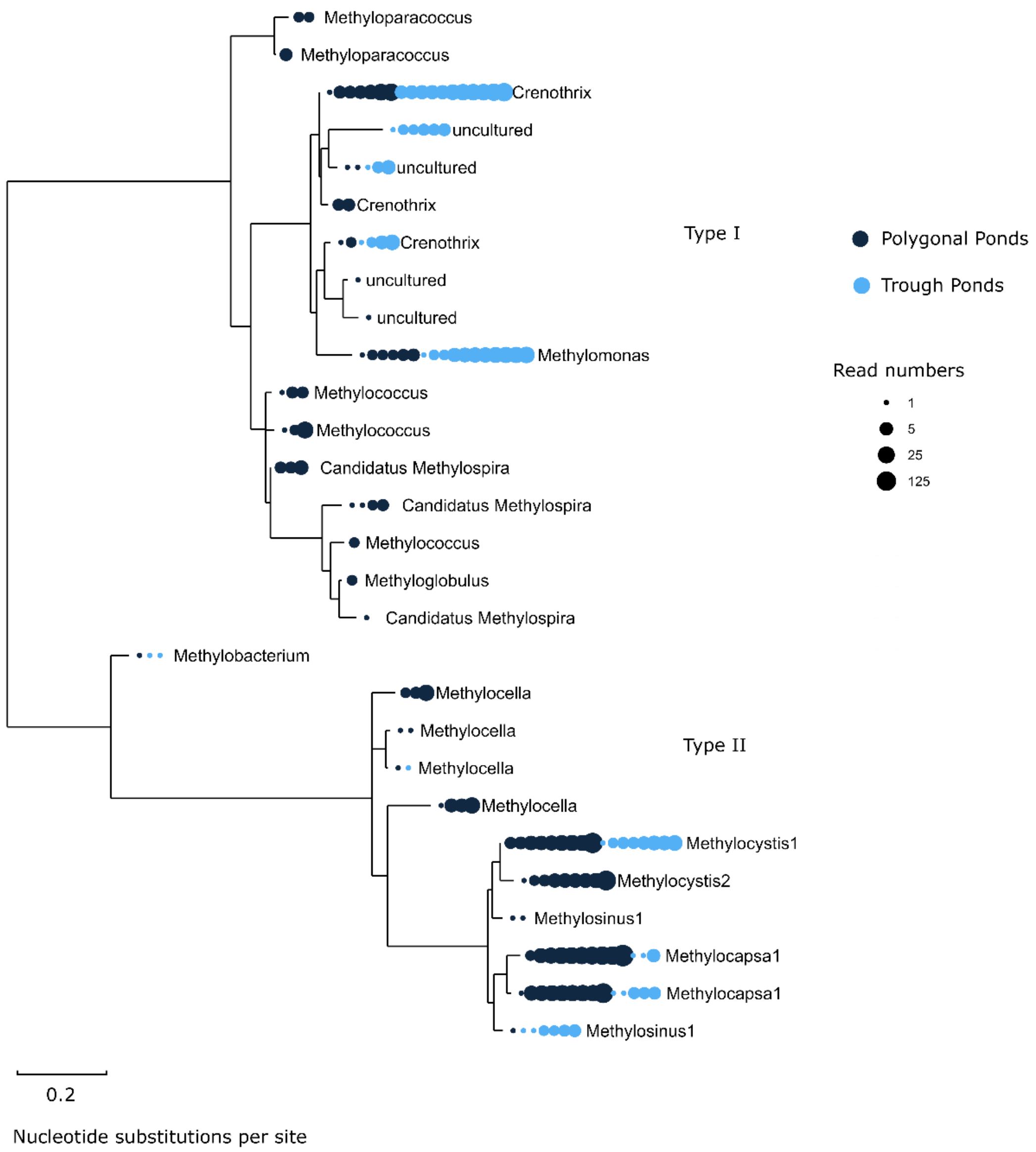

Figure S4: Phylogenetic relationship of methanotrophs (Maximum Likelihood method) and their relative abundance in each pond. Each circle represents one pond.

Table S2: Average read numbers of OTUs that significantly contributed to the difference of the eukaryotic microbial communities between polygonal and trough ponds (simper analysis). Only OTUs with a contribution >1 % are shown. Distance: % difference to closest know sequence (accession number).

OTUs	Polygonal	Trough	% contribution	p-value	Accession Number	Distance (%)	Phylum	Species
OTU 1	93.35	593.4	20.97	0.001	AM051191	0	Cryptophyta	*Crytomonas marssonii*
OTU 5	222.95	0.05	8.23	0.001	JF730783	0	Ochrophyta	*uncultured Uroglena*
OTU 6	161.15	3.85	5.89	0.001	GU067811	2.9	Chlorophyta	*uncultured Monomastix*
OTU 7	0.5	141.95	5.24	0.001	EF014365	15.2	Blastocladiomyco	*Catenaria anguillulae*
OTU 4	0.65	138.2	5.09	0.001	GU067821	0	Chlorophyta	*uncultured Chlamydomonadales*
OTU 10	117.15	1.85	4.29	0.001	KC861672	0	Chlorophyta	*Coelastrella ellipsoidea*
OTU 11	0	53.35	1.97	0.001	EF100355	6.3	Ochrophyta	*uncultured Paraphysomonas*
OTU 14	51.1	0	1.88	0.001	HM135075	0.3	Chlorophyta	*uncultured Monomastix*
OTU 22	1.3	46.85	1.72	0.007	FR865749	0	Chlorophyta	*Chaetophora incrassata*
OTU 12	7.25	43.6	1.61	0.04	AB491653	5.6	Chlorophyta	*Monomastix minuta*
OTU 13	41.5	0.05	1.53	0.001	GU067985	0	Charophyta	*uncultured Chaetosphaeridium*
OTU 17	33.85	0.05	1.25	0.018	HAFO01005448	2.9	Ochrophyta	*Uroglena WA34KE*
OTU 21	33.2	0	1.22	0.001	AB771832	4.7	Ochrophyta	*unciltered Chrysophyceae*
OTU 16	31.05	0.1	1.14	0.004	EF443138	7.1	Chytridiomycota	*Clydaea vesicula*
OTU 18	29.6	1.25	1.09	0.005	HM135093	4.6		*unciltered Bicosoecida*
OTU 29	28.75	0	1.06	0.001	HF549063	5.8	Ochrophyta	*Tesselaria lapponica*

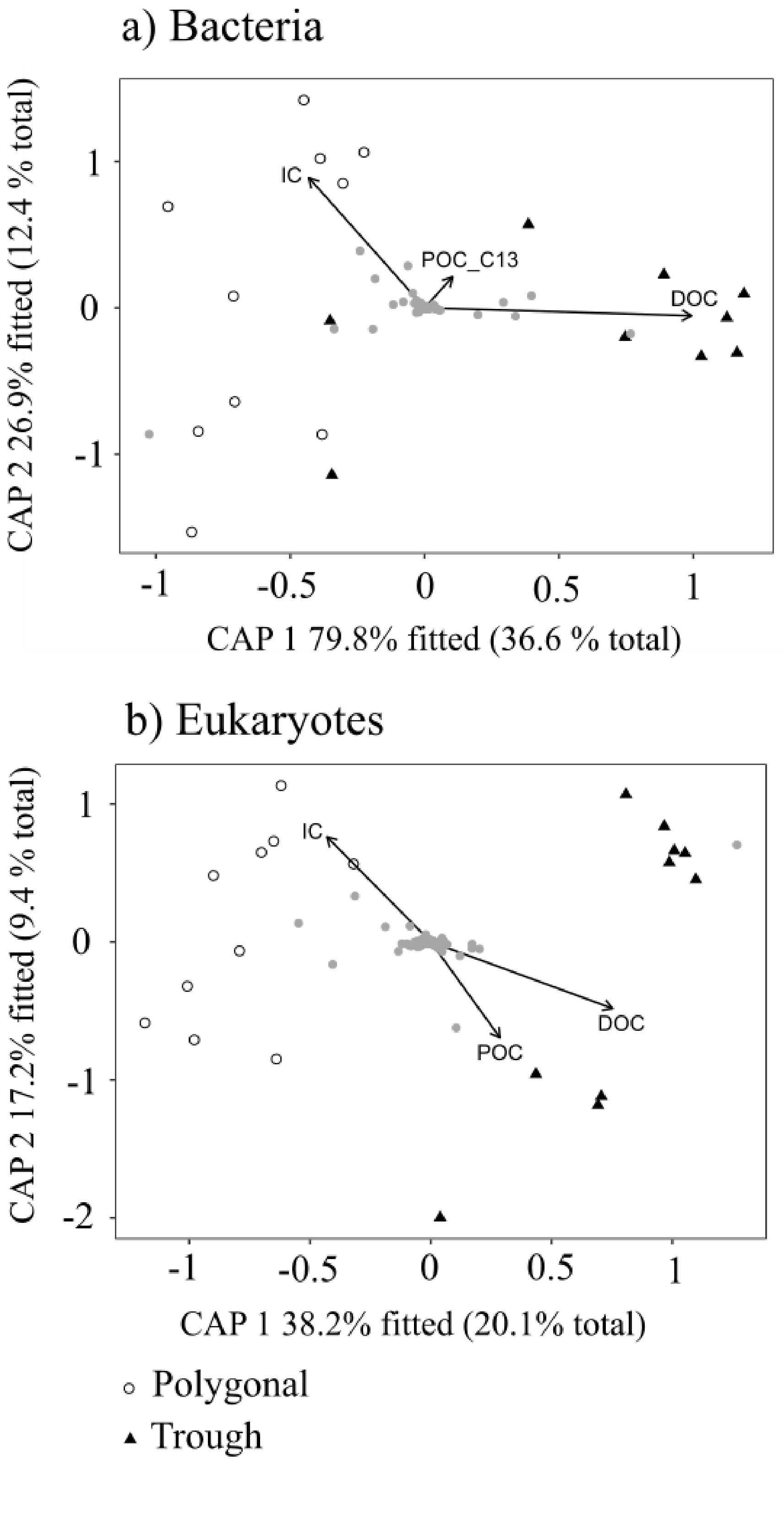

Figure S5: db-RDA of the a) bacterial b) micro-eukaryotic communities. Shaded points represent the OTUs and arrows represent significant environmental parameters. On the axis, the percentage of inertia is represented for the fitted model and for the total inertia. Pond B05 was excluded from the analysis of the bacterial communities.

a)

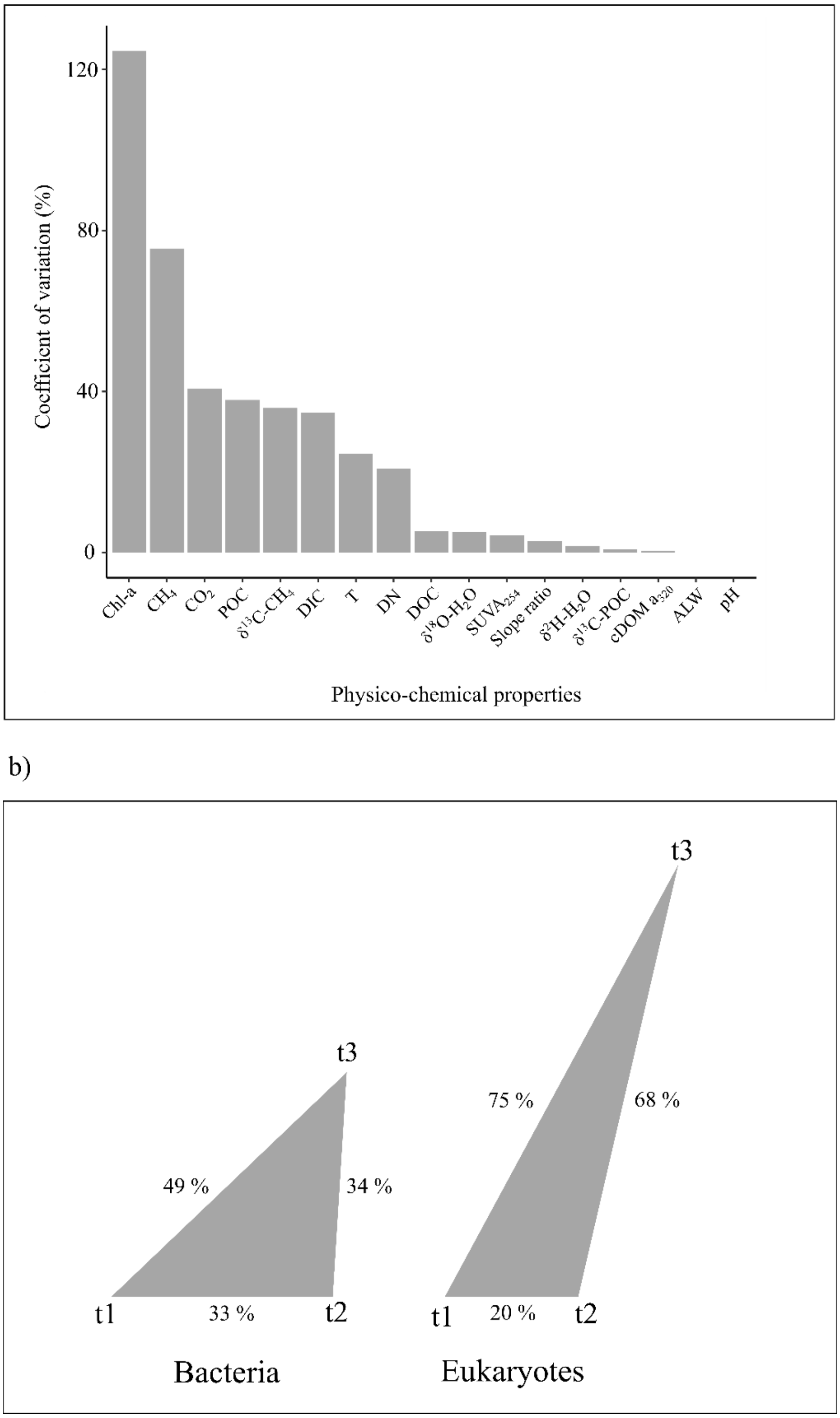

Figure S6: a) Coefficient of variation of the physico-chemical properties in pond B01 from the three sampling dates: t1: 13/07/2018, t2: 17/07/2018, t3: 23/07/2018. Abbreviations are described in Table 1. b) Bray-Curtis dissimilarity for the prokaryotic and eukaryotic communities for the 3 sampling dates.
